# Infrared Irradiation‐Assisted Sonogashira Cross‐Coupling Reaction

**DOI:** 10.1002/cssc.71003

**Published:** 2026-08-25

**Authors:** Ambra M. Fiore, Alessia Damato, Pietro Cotugno, Angela Punzi, Gianluca M. Farinola

**Affiliations:** ^1^ Dipartimento di Chimica Università degli Studi di Bari Aldo Moro Bari Italy

**Keywords:** catalyst recycle, green approach, infrared irradiation, Sonogashira, sustainability

## Abstract

A sustainable and energy‐efficient protocol for the Pd‐catalyzed Sonogashira cross‐coupling reaction is reported, which is promoted by infrared (IR) irradiation in aqueous media with a recyclable Pd/C catalyst. The methodology enables C(sp^2^)─C(sp) bond formation under mild conditions with low catalyst loading (1 mol% Pd), affording the coupling products in good to excellent yields from a broad range of aryl iodides, including substrates bearing electron‐donating and electron‐withdrawing groups, and various terminal alkynes. The use of water as a benign reaction medium, combined with IR irradiation as an alternative energy input, contributes to improved sustainability and shorter reaction times compared to conventional thermal heating, while minimizing the formation of undesired by‐products. Notably, the heterogeneous Pd/C catalyst can be easily recovered and reused over several cycles, showing only a slight loss of activity, thus enhancing the overall process sustainability, after the second cycle.

## Graphical abstract

The Pd‐catalyzed Sonogashira cross‐coupling was developed under sustainable conditions using IR irradiation in water and a recyclable Pd/C catalyst. The protocol promotes efficient C(sp^2^)─C(sp) bond formation under mild conditions with low Pd loading (1 mol%), providing good to excellent yields for various aryl iodides and terminal alkynes. This protocol improves sustainability reduces reaction times, and limits by‐product formation.

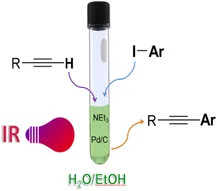

## 1 Introduction

Palladium‐catalyzed cross‐coupling reactions represent one of the most powerful tools for the construction of carbon–carbon bonds in modern organic synthesis and are widely employed in the preparation of fine chemicals and biologically active molecules [1]. Among these transformations, the Sonogashira cross‐coupling reaction is one of the most valuable methods for the formation of C(sp^2^)─C(sp) bonds through the coupling of aryl or vinyl halides with terminal alkynes, providing access to internal alkynes that are key structural motifs in pharmaceuticals, natural products, and functional materials [2, 3]. The classical Sonogashira protocol typically involves a palladium catalyst in combination with a copper co‐catalyst, which participates in the transmetalation step with the palladium complex and facilitates the catalytic cycle [4]. However, the presence of copper may promote undesired side reactions, such as Glaser‐type oxidative homocoupling of terminal alkynes, leading to the formation of diyne by‐products and reducing the overall selectivity of the process [5]. For these reasons, considerable efforts have been dedicated in recent years to the development of more sustainable copper‐free Sonogashira protocols, particularly employing heterogeneous palladium catalysts. Although these systems often allow catalyst recovery and reuse, many reported procedures still suffer from limitations such as long reaction times, relatively high temperatures, or the use of hazardous solvents such as DMF, THF, or acetonitrile [6].

To address the limitations of these protocols, which remain distant from the principles of green and sustainable chemistry, several studies have been directed toward the development of alternative methods. Among them, the use of amines acting both as bases and the reaction media [7], aqueous solvent systems such as DMF/H_2_O in Pd/C‐catalyzed transformations [8], and surfactant‐assisted aqueous conditions have been investigated [9]. Although these approaches have significantly improved the sustainability of the Sonogashira reaction, important limitations remain, including prolonged reaction time [10] and the need for relatively high palladium loading (Pd > 2 mol%). In recent years, considerable efforts have been devoted to the development of advanced heterogeneous catalysts specifically designed for aqueous‐phase Sonogashira coupling [11, ‍12]. Among the most promising examples are single‐atom Pd catalysts embedded in mesoporous organosilica frameworks, which suppress metal aggregation while enhancing catalyst stability in water [13] and Pd catalysts encapsulated within cross‐linked ethylcellulose micro/nanocapsules, which facilitate catalyst recovery and minimize metal contamination [14]. Similarly, palladium nanoparticles immobilized on biopolymers, cellulose‐based materials, and metal–organic frameworks have demonstrated the benefits of catalyst immobilization in improving catalyst recyclability and sustainability [15]. Despite the remarkable advances, these catalytic systems generally rely on elaborated support architecture or multistep preparation procedures, limiting their practical accessibility. Therefore, the development of simple, commercially available heterogeneous catalysts capable of operating efficiently under environmentally benign conditions remains highly desirable. In parallel, alternative external activation methods to improve the reaction efficiency have attracted increasing interest. In this context, microwave irradiation [16, 17], mechanical milling [18, 19, 20], and photocatalytic systems have been considered as alternative activation reaction methods that can replace conventional thermal heating and enhance overall reaction efficiency [21]. Infrared (IR) irradiation provides a more straightforward and accessible activation pathway method with respect to these technologies, which require specialized and expensive instruments. IR activation represents a cheaper alternative to enable rapid and efficient energy transfer to the reaction medium and to significantly accelerate chemical transformations [22, 23]. Among the available IR sources, near‐infrared (NIR) emitters, such as incandescent light bulbs equipped with a tungsten filament sealed in a quartz envelope filled with a halogen gas and exhibiting an emission centered at 1.2 μm, are particularly attractive due to their fast response time and high energy efficiency, as they convert a large fraction of the input energy into IR radiation and enable rapid heating of the reaction mixture [24, 25, 26]. IR irradiation has already been used to promote various organic transformations, including condensations, oxidations, and heterocycle synthesis, with good results for selectivity and reduced reaction times, although the mechanistic origin of this effect has not been fully investigated so far. However, only a limited number of palladium‐catalyzed cross‐coupling reactions promoted by IR irradiation have been reported to date, including Mizoroki–Heck, Suzuki–Miyaura, direct arylation, and Buchwald–Hartwig amination reactions [27, 28, 29, 30, 31, 32, 33]. In the frame of our studies on the development of sustainable Pd‐catalyzed protocols [34, 35, 36, 37, 38], we report herein the first example of an IR‐assisted Pd‐catalyzed copper‐free Sonogashira cross‐coupling reaction. In addition to using IR irradiation as a convenient reaction mode, our reactions are carried out using green solvents such as water and ethanol, without the exclusion of an air atmosphere and without copper additives, enabling the rapid and sustainable synthesis of internal alkynes.

## 2 Results and Discussion

To optimize the reaction conditions, the coupling of ethynylbenzene (**1a**) with iodobenzene (**2a**) to afford product **3a** was selected as a model reaction (Table 1). Reactions were performed using an excess of alkyne (1.4 equiv), a commercially available palladium catalyst, and a base (3.6 equiv) in various environmentally benign solvents under IR irradiation. Reactions were carried out in a simple setup consisting of a Carius tube in which all reagents were introduced under air. The glass reactor was placed at a distance of 4 cm from a NIR lamp (λ_max ≈ 1.2 µm; see Supporting Information for details). As an initial test, the Sonogashira coupling in a H_2_O/EtOH mixture (5 mL) using Pd/C (5 mol%) led to complete conversion within 2 h (Table 1, entry 1). In contrast, Cu‐ or Cu/Pd‐based systems were markedly less effective: CuO (10 mol%) afforded only limited product formation (20%, Table S1, entry 1), and similarly low conversions were obtained using CuO in combination with Pd/C (40%, Table S1, entry 2). CuI in combination with Pd/C also showed poor activity under these conditions (≈30% conversion, Table S1, entry 3). Since complete conversion was achieved within 2 h using 5 mol% Pd/C (entry 1), we next investigated whether the catalyst loading could be reduced while maintaining full conversion within a shorter reaction time. Accordingly, the reaction was performed for 1 h using 2.5 mol% Pd/C (entry 2), and complete conversion was still observed. Further lowering the catalyst loading to 2–1 mol% still resulted in full conversion (entries 3–4), whereas decreasing it to 0.5 mol% led to a noticeable drop in efficiency, with a conversion of ca. 87% (entry 5). Reducing the irradiation time to 30 min resulted in only a slight decrease in conversion (≈90%, entry 6), as well as reducing the reaction solvent volume to 1 mL (entry ‍7). In contrast, the use of water as the sole solvent completely suppressed the reaction (entry 8). Similarly, using ethanol as the only solvent resulted in a reduced conversion of 71% (entry 9), whereas employing a green solvent with a low environmental impact, such as cyclopentyl methyl ether (CPME), resulted in poor conversion (≈10%, entry 10). A solvent‐free condition was also evaluated; however, the formation of numerous undesired by‐products was observed (entry 11). Among the other protic solvents investigated, methanol afforded complete conversion but promoted significant by‐product formation (entry 12), while isopropanol showed a slightly lower conversion comparable to that obtained with ethanol alone (entry 13 vs. entry 9). Finally, the effect of varying the water‐to‐ethanol ratio was examined. Increasing the water content relative to the optimized 1:1 mixture maintained complete conversion but favored the formation of undesired by‐products (entries 14 and 15), whereas increasing the ethanol fraction progressively decreased the conversion (entries 16 and 17). Overall, these results demonstrate that the 1:1 water/ethanol mixture provides the best compromise between reactivity and selectivity, suggesting a synergistic effect between the two solvents. Previous experimental and theoretical studies have highlighted the potential beneficial role of water in Sonogashira reactions [39, 40], and recently, water has been shown to directly influence the catalytic cycle by facilitating the conversion of the π‐coordinated alkyne complex into the corresponding σ‐alkynyl palladium complex, thereby lowering the barrier for C─H activation [41]. Based on our results, we hypothesize that the superior performance of the water/ethanol mixture arises from the combination of several factors. Besides improving the reaction medium, water may promote “on‐water” effects that increase the local concentration of the reactants through the formation of microscopic organic droplets [42], enhance the stabilization of ionic species such as the ammonium salts generated during the reaction [43], and facilitate key steps of the catalytic cycle. The optimal 1:1 water/ethanol ratio likely provides the best balance between these effects and the enhanced solubility of the organic substrates, as ensured by ethanol.

**TABLE 1:** Optimization of the reaction conditions.^a^

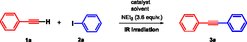				
Entry	Catalyst	Solvent	Time, h	**Conversion** b
1	Pd/C (5 mol%)	H_2_O:EtOH (5 mL)	2	100% (87%)^c^
2	Pd/C (2.5 mol%)	H_2_O:EtOH (2 mL)	1	100% (91%)^c^
3	Pd/C (2 mol%)	H_2_O:EtOH (2 mL)	1	100% (85%)^c^
4^d^	**Pd/C (1 mol%)**	**H**_**2**_ **O:EtOH** **(2 mL)**	**1**	**100% (90%)** c
5	Pd/C (0.5 mol%)	H_2_O:EtOH (2 mL)	1	87%
6	Pd/C (1 mol%)	H_2_O:EtOH (2 mL)	30 min	90%
7	Pd/C (1 mol%)	H_2_O:EtOH (1 mL)	1	92%
8	Pd/C (2.5 mol%)	H_2_O (2 mL)	1	0%
9	Pd/C (1 mol%)	EtOH (2 mL)	1	71%
10	Pd/C (2 mol%)	CPME (2 mL)	1	10%
11	Pd/C (1 mol%)	Solvent‐free	1	76%
12	Pd/C (1 mol%)	MeOH	1	>99^e^
13	Pd/C (1 mol%)	iPrOH	1	85
14	Pd/C (1 mol%)	H_2_O/EtOH (0.75:0.25)	1	>99^e^
15	Pd/C (1 mol%)	H_2_O/EtOH (0.625:0.375)	1	>99^e^
16	Pd/C (1 mol%)	H_2_O/EtOH (0.375:0.625)	1	83
17	Pd/C (1 mol%)	H_2_O/EtOH (0.25:0.75)	1	75
18^f^	Pd/C (1 mol%)	H_2_O:EtOH (2 mL)	1	50%
19^f^	Pd/C (2 mol%)	H_2_O:EtOH (2 mL)	1	88%

*Note:* The reaction conditions highlighted in bold correspond to the optimized reaction conditions.

a Unless otherwise stated, the reactions were performed using NEt_3_ (3.6 equiv.), **1a**:**2a** (1.4:1 mmol), under IR irradiation as detailed in the table. In all water/ethanol mixtures, a 1:1 volume ratio was used.

b Conversion determined by GC analysis on the crude product.

c Yields on isolated products (column chromatography).

d To demonstrate the scalability of the protocol, the reaction was also performed on a 10 mmol scale (based on **2a**), affording the desired product in an essentially identical isolated yield (89%) to that obtained on the 1 mmol scale (90%).

e For these reactions, the formation of by‐products was observed.

f The reaction was performed under conventional thermal heating in a pre‐heated oil bath at 110 °C for 1 h (110 °C corresponds approximately to the reaction temperature under IR irradiation measured with an internal thermometer).

Alternative palladium heterogeneous catalysts showed comparable but slightly lower performance (93%–95%, Table S1, entries ‍6–7). We also evaluated a Pd(II) source (Table S1, entry ‍8) to investigate whether it could undergo in situ reduction to Pd(0) under our reaction conditions and effectively catalyze the Sonogashira coupling. As shown in Table S1, entry ‍8, the conversion was only slightly lower (92%) than the optimized conditions, indicating that the catalytic system is indeed compatible with the Pd(II) precursor. Moreover, since Pd(OAc)_2_ is more expensive than Pd/C and lacks the advantage of recyclability, Pd/C was chosen as the optimal catalyst for this transformation.

A control experiment carried out in the absence of the catalyst showed that no reaction occurred, confirming the essential role of the Pd catalyst in promoting the reaction (Table S1, entry ‍9). Finally, the model reaction was performed under conventional thermal conditions (oil bath, 110 °C), corresponding to the temperature reached under IR irradiation. Under thermal conditions, the reaction reached 50% conversion after 1 h (Table 1, entry 18) and was accompanied by the formation of a *trans*‐enyne by‐product, which was not detected under IR irradiation (Scheme ‍1). After 1 h, *trans*‐enyne (Scheme ‍1, product **4**) was identified as the major product, whereas the desired product **3a** was formed as the minor one (**4**:**3a** ratio = 1.4:1). Since both reagents were still present at this stage, the reaction was further monitored over longer reaction times to investigate the product evolution. After 2 h, the product distribution remained essentially unchanged; after 3 h, the amount of *trans*‐enyne increased, while the relative abundance of the desired product decreased (**4**:**3a** ratio = 2.2:1). These data suggest that *trans*‐enyne formation originates from a secondary transformation of the initially formed coupling product, in agreement with the literature reports [44, 45]. The absence of *trans*‐enyne formation under IR irradiation could be attributed to the faster reaction progress observed under these conditions. Actually, the rapid consumption of phenylacetylene minimizes the exposure time of the Sonogashira product to the reaction conditions, thus limiting subsequent transformations. In contrast, the slower reaction rate observed under conventional thermal heating allows sufficient time for *trans*‐enyne formation. Consistently, increasing the Pd loading (2 mol%, entry 19) under thermal conditions accelerates the coupling process and reduces *trans*‐enyne formation (**4**:**3a** ratio = 1:4.2), further supporting the correlation between the reaction rate and suppression of the undesired pathways. Overall, these observations are consistent with previous studies indicating that faster conversion rates can effectively minimize consecutive secondary transformations [46].

**SCHEME 1:**
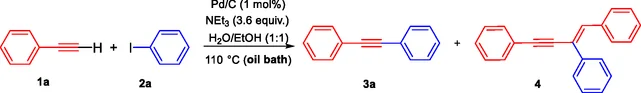
Sonogashira cross coupling under conventional thermal heating.

The reaction under the optimized conditions was also evaluated using different phenyl halides, such as bromobenzene and chlorobenzene. As expected, no product formation was observed in the case of chlorobenzene, reflecting its low reactivity (Table S1, entry 4). In contrast, bromobenzene afforded the desired coupling product; however, the reaction proceeded slowly, reaching only 40% conversion after 3 h, indicating significantly lower reactivity compared to iodobenzene (Table S1, entry 5). To further demonstrate the practical synthetic utility of this mild, air‐tolerant, and environmentally friendly protocol, the model Sonogashira coupling between **1a** and **2a** was performed on a 10 mmol scale under the optimized reaction conditions reported in Table 1, entry 4. The scale‐up experiment proceeded smoothly, affording the desired product in an 89% isolated yield (Table 1, note d), which is comparable to the yield obtained on the 1 mmol scale (90%, Table 1, entry ‍4). These results demonstrate the excellent scalability and robustness of the protocol, which maintains high catalytic performance and reaction efficiency upon scale‐up.

**SCHEME 2:**
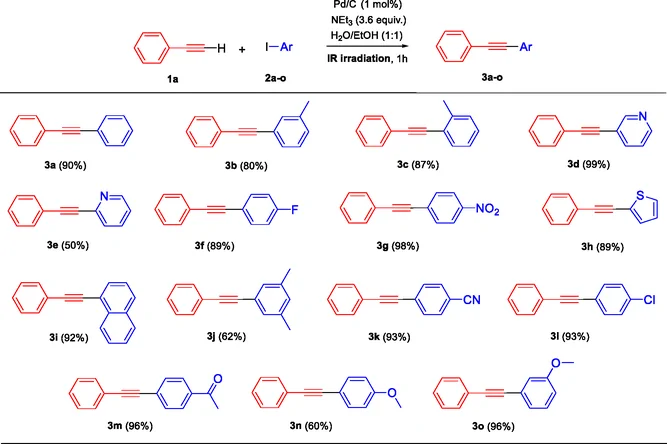
Scope of aryl halide in Sonogashira C─C cross‐coupling reactions. Reaction conditions: phenylacetylene **1a** (1.4 mmol), aryl halide **2a–o** (1 mmol), Pd/C (1 mol%), triethylamine (3.6 mmol), H_2_O and EtOH (1:1, v/v, 2 mL), IR irradiation, under air, 1 h. Product yields reported are determined after chromatographic purification; full experimental details and procedures are provided in the Supporting Information.

Having thus identified the optimal reaction conditions, a variety of aryl iodides were investigated, and the results of this screening are summarized in Scheme 2. Overall, the protocol proved to be broadly applicable, affording good to excellent yields across a wide range of aryl iodides bearing both electron‐withdrawing and electron‐donating substituents. Substrates containing electron‐withdrawing groups, such as fluorine (**3f**: 89%), nitro (**3g**: 98%), cyano (**3k**: 93%), chlorine (**3l**: 93%), and acetyl (**3m**: 96%) substituents, generally provided excellent yields. Notably, comparable efficiency was also observed with several electron‐donating substituents, including both weak electron‐donating groups, as demonstrated by compounds **3b** (80%), **3c** (87%), and **3j** (62%), and strongly electron‐donating groups, as for **3n** (60%) and **3o** (96%). Heteroaromatic and polycyclic aromatic hydrocarbons were also well tolerated; in particular, pyridyl (**3d**: 99% and **3e**: 50%), thienyl (**3h**, 89%), and naphthyl (**3i**, 92%) derivatives were obtained in good to excellent yields.

**SCHEME 3:**
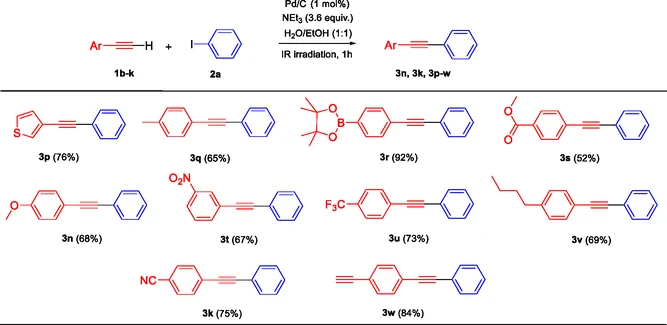
Scope of alkynes in Sonogashira C─C cross‐coupling reactions.

The scope of the reaction with respect to the alkyne component was also investigated (Scheme 3). Overall, the developed protocol displayed broad substrate compatibility, delivering the desired products in good to excellent yields across a variety of functionalized aryl alkynes. Electron‐deficient substrates bearing methyl ester (**3s**: 52%), nitro (**3t**: 67%), trifluoromethyl (**3u**: 73%), and cyano (**3k**: 75%) substituents were efficiently converted, affording the corresponding products in satisfactory to good yields. Additionally, alkynes featuring electron‐neutral and donating groups, including methyl (**3q**: 65%), butyl (**3v**: ‍69%), and methoxy (**3n**: 68%) substituents, were well tolerated, providing consistently good results. Particularly remarkable is the excellent performance of the methodology with synthetically valuable boronate functionalities, which also offer opportunities for subsequent derivatization (**3r**: 92%). The protocol also proved effective with heteroaromatic substrates, as exemplified by the thiophene derivative, which was isolated in a 76% yield (**3p**, Scheme 3). The methodology was further evaluated using 2,4‐diethynylbenzene as the alkyne coupling partner, affording the corresponding monoarylated product **3w** in an 84% yield, which represents a potential intermediate in the synthesis of unsymmetrically substituted oligo(phenylene ethynylene) derivatives [47].

Finally, the applicability of the method to aliphatic alkynes was assessed; however, no product formation was observed in this case. This result can be attributed to the copper‐free protocol since copper is generally considered essential for the activation of aliphatic alkynes in Sonogashira reactions [48, 49].

**FIGURE 1:**
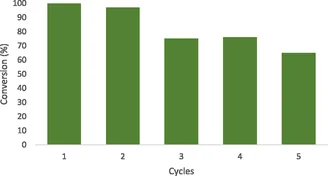
Recycling test of the Pd/C‐catalyzed Sonogashira coupling, performed under the optimized conditions for the model reaction.

The recyclability of the heterogeneous catalyst was evaluated using the model reaction, as reported in Table 1. After 1 h, the reaction mixture was analyzed to determine the conversion of the substrate into the desired product. The reaction mixture was then centrifuged to recover the Pd/C catalyst, allowing the separation of the catalyst from the organic phase. The recovered catalyst was washed five times with ethanol, after which fresh reagents and solvents were added, and the reaction was performed under the same conditions. The catalyst could be reused for five consecutive runs (Figure 1); however, a gradual decrease in conversion was observed after the second cycle.

Based on these results, a palladium‐leaching experiment was performed to investigate the origin of the decrease in catalytic activity upon recycling. The model reaction was carried out and stopped after 10 min, reaching a 70% conversion. The reaction mixture was then hot‐filtered through a Teflon filter (0.2 μm) to remove the solid catalyst, and the resulting filtrate was subjected to the same reaction conditions under IR irradiation for an additional 50 min. After a total reaction time of 1 h, the final reaction mixture was analyzed, showing a conversion of approximately 85%. The formation of additional products after the filtration test indicates that a small amount of palladium species leaches into the solution. Therefore, the decrease in conversion observed after the second catalytic cycle can be attributed to the leaching of active palladium species into the reaction medium.

To evaluate the catalytic efficiency of the supported palladium catalyst, the turnover number (TON) and turnover frequency (TOF) values for the Pd/C system were calculated based on the total Pd loading and reaction time (Table 2). These values were compared with those obtained for the other heterogeneous palladium catalysts investigated in this study. Under IR irradiation, Pd/C showed the highest catalytic productivity, providing a TON and TOF of 100 after 1 h (Table 2, entry 1). Similar performances were observed for Pd/BaSO_4_ and Pd/AlO(OH), with TON/TOF values of 95 and 93, respectively (Table 2, entries 2 and 3). The recyclability of Pd/C was assessed by calculating the cumulative TON and average TOF over five consecutive cycles. The catalyst reached a cumulative TON of 413 with an average TOF of 82.6 h^−1^. The catalytic performance of Pd/C was then compared with representative heterogeneous palladium catalysts reported for Sonogashira coupling. Although higher TON and TOF values have been achieved using engineered systems based on water‐soluble phosphine ligands, hybrid Pd/Cu supports, or modified Pd/C catalysts [50, 51, 52, 53], direct comparison is complicated by the substantial differences in catalyst design. Nevertheless, the cumulative TON of 413 and average TOF of 82.6 h^−1^ obtained with a commercially available, unmodified Pd/C catalyst demonstrate an attractive balance between catalytic efficiency, operational simplicity, and recyclability without requiring complex catalyst preparation or post‐synthetic modification.

**TABLE 2:** Comparison of the Sonogashira reaction between iodobenzene and phenylacetylene, catalyzed by various catalysts.^a^

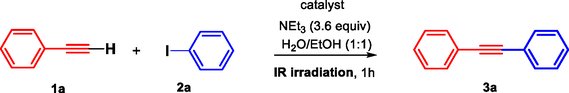					
Entry	Catalyst	Time, h	**Conversion, %** b	**TON** c	**TOF, h** **−1** d
1	Pd/C	1	>99	100	100
2	Pd/BaSO_4_	1	95	95	95
3	Pd/AlO(OH)	1	93	93	93

a Reagents and conditions: phenylacetylene (1.4 mmol), iodobenzene (1.0 mmol), NEt_3_ (3.6 mmol), in H_2_O/EtOH (1:1, 2 mL).

b Conversion determined by GC analysis on the crude product.

c TON, turnover number (mmol of selectively formed product/mmol of Pd catalyst).

d TOF, turnover frequency (TON/time).

## 3 Conclusion

To the best of our knowledge, our report represents the first example of IR‐irradiation‐assisted Sonogashira coupling carried out in a green solvent system (water/ethanol) under nonanhydrous conditions. The developed protocol demonstrates broad functional group tolerance and is applicable to a wide range of iodoarenes and substituted alkynes, representing a general and sustainable approach for the formation of C(sp^2^)─C(sp) bonds. A key feature of this method is the combined use of IR irradiation and a heterogeneous catalytic system, which enables efficient reaction promotion under mild conditions. Notably, the process proceeds with high selectivity, minimizing the formation of undesired by‐products. From a sustainability perspective, the protocol aligns well with the principles of green chemistry, not only due to the use of environmentally benign solvents but also because of the recyclability of the catalyst. The catalytic system was successfully reused for five consecutive cycles, with a slight decrease in reaction yield after the second cycle. Overall, these findings provide compelling evidence for the effectiveness of IR‐assisted catalysis in organic synthesis, offering a new strategy for achieving efficient, selective, and sustainable cross‐coupling reactions. Our study opens new possibilities for the development of IR irradiation‐assisted synthetic protocols, contributing to the advancement of greener and more efficient catalytic processes.

## Conflicts of Interest

The authors declare no conflicts of interest.

## Supporting information

The authors have cited additional references within the Supporting Information [54, 55, 56, 57, 58, 59, 60, 61].

## Data Availability

Additional data supporting the findings of this study are available in the supporting information of this article.
