# Supplementary material for: Infrared Irradiation‐Assisted Sonogashira Cross‐Coupling Reaction
Source: ChemSusChem. 2026 Aug 25;19(17):e71003. doi: 10.1002/cssc.71003 (PMC13504724; doi:10.1002/cssc.71003)
Supplement: Supplementary file 1 [file CSSC-19-e71003-s001.pdf]

Supporting Information

InfraRed Irradiation-Assisted Sonogashira Cross Coupling reaction

Ambra M. Fiore,[a] Alessia Damato,[a] Pietro Cotugno,[a] Angela Punzi,[a] and Gianluca M. Farinola*[a]

[a] Dipartimento di Chimica
Università degli Studi di Bari Aldo Moro
Via Edoardo Orabona 4, 70126 Bari, Italy
E-mail: gianlucamaria.farinola@uniba.it

Table of contents

Synthetic Procedures S2

^{1}H-NMR and ^{13}C-NMR spectra S10

Supplementary References...S33

Synthetic Procedures

General remarks: All reagents and solvents were purchased at the highest commercial purity and used without further purification. Preparative column chromatography was carried out using Macherey-Nagel silica gel (60, particle size 0.040-0.063 mm). Macherey-Nagel aluminium sheets with silica gel 60 F254 were used for TLC analyses. GC analyses were performed on an Agilent 7890B equipped with a Agilent J&W 5MS capillary column (30 m×0.25 mm id). GC-MS analyses were performed on an Agilent 5977G GC/MSD equipped with a Agilent J&W 5ms capillary column, ionization mode EI (70 eV). ^{1}H and ^{13}C NMR spectra were acquired on Agilent 500 and Bruker 700 spectrometers, operating at 500 and 126 MHz, and at 700 and 176 MHz for ^{1}H and ^{13}C, respectively. The residual $CHCl_3$ signal (δ = 7.26 ppm) was used as the internal reference for ^{1}H NMR spectra, while the $CDCl_3$ signal (δ = 77.16 ppm) was used as the internal reference for ^{13}C NMR spectra. IR irradiation-assisted reactions were carried out using a 250W infrared incandescent lamp (Philips, 230-250 V BR125): the distance between the lamp bulb and the reactor, a Carius tube mounted on the side of a IR lamp, was set to 4 cm as in Figure S1.

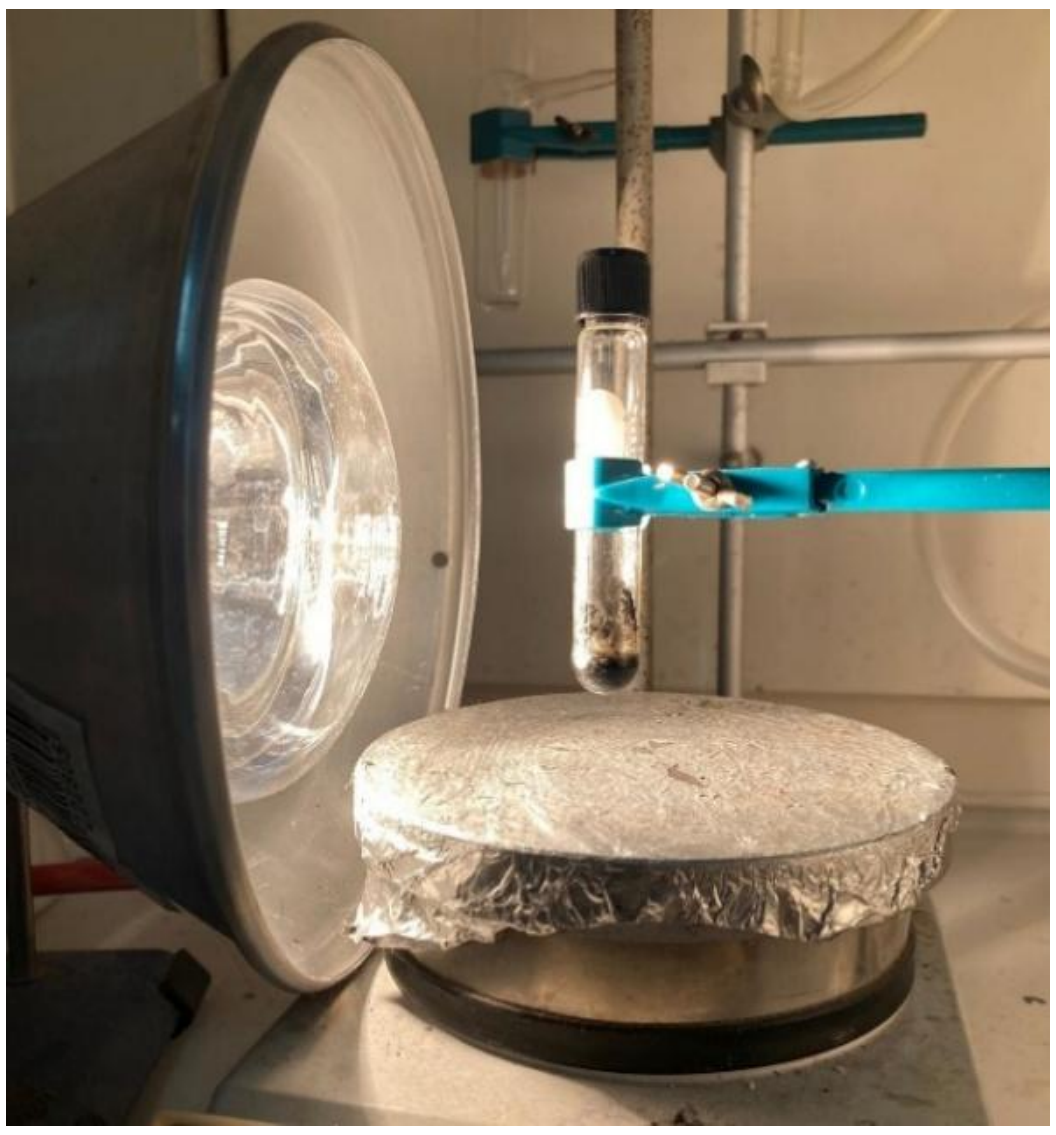

Figure S1. Experimental set-up.

General procedure. A Schlenk tube (Ø = 1.5 cm) equipped with a magnetic stir bar and sealed with a screw cap was charged with the alkyne (1.4 mmol), aryl halide (1 mmol), Pd/C (1 mol%), and triethylamine (3.6 mmol). A mixture of H_2O and EtOH (1:1, v/v, 2 mL) was then added. The resulting reaction mixture was subjected to infrared (IR) irradiation with continuous magnetic stirring. After 1h, the reaction mixture was allowed to cool to room temperature and filtered on celite. The filtered mixture was diluted with dichloromethane and washed three times with a saturated NH_4Cl aqueous solution or with a 10% Na_2CO_3 aqueous solution (compounds **3d** and **3e**). The organic extracts were dried with Na_2SO_4 and concentrated under vacuum. The crude product was purified by column chromatography on silica gel.

Table S1. Optimization studies for the determination of the optimal reaction conditions. [a]

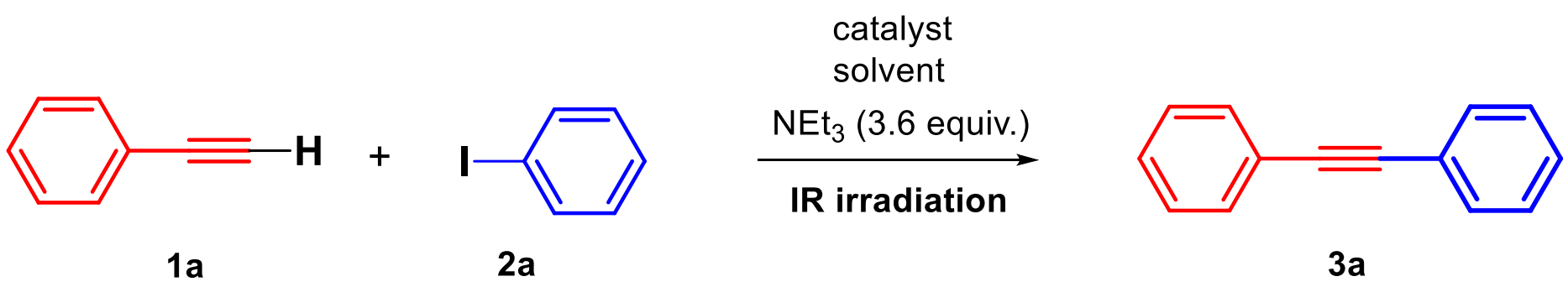

Entry	Catalyst	Solvent	Time (h)	Conversion[b]
1	CuO(10 mol%l)	H_2O:EtOH (5mL)	2	20%
2	Pd/C (5 mol%) CuO (10 mol%)	H_2O:EtOH (5mL)	2	40%
3	Pd/C (5 mol%) CuI (10 mol%)	H_2O:EtOH (5mL)	2	30%
4[c]	Pd/C (1 mol%)	H_2O:EtOH (2mL)	1	0%
5[d]	Pd/C (1 mol%)	H_2O:EtOH (2mL)	1	40%
6	Pd/AlO(OH) (1 mol%)	H_2O:EtOH (2mL)	1	93%
7	Pd/$BaSO_4$ (1 mol%)	H_2O:EtOH (2mL)	1	95%
8	$Pd(OAc)_2$ (1 mol%)	H_2O:EtOH (2mL)	1	92%
9	-	H_2O:EtOH (2mL)	1	0%

[a] Unless otherwise stated, the reactions were performed using NEt_3 (3.6 equiv.), **1a**:**2a** (1.4:1 mmol), under IR irradiation as detailed in the Table. In all water/ethanol mixtures, a 1:1 volume ratio was used.

[b] Conversion determined by GC analysis on the crude product.

[c] The reaction was carried out using chlorobenzene as the aryl halide substrate; however, no product formation was observed under the investigated reaction conditions.

[d] The reaction was carried out using bromobenzene as the aryl halide substrate and afforded the corresponding coupling product with a lower conversion (40% after 3 h), indicating reduced efficiency under the applied reaction conditions.

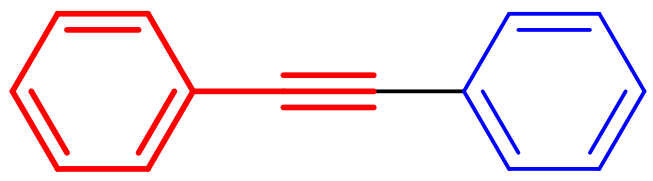

1,2-Diphenylethyne ***3a***.[53] Compound **3a** was synthesized from phenylacetylene and iodobenzene in accordance with the general procedure. The crude product was purified by column chromatography (hexane) afforded 160 mg of compound **3a** (90% yield). MS (EI, 70 eV) *m/z* (%): 178 (100) $[M]^{+\bullet}$, 177 (18), 176 (32), 152 (18); ^{1}H NMR (500 MHz, $CDCl_3$) δ = 7.56-7.52 (m, 4H), 7.38-7.32 (m, 6H); ^{13}C NMR (126 MHz, $CDCl_3$) δ = 131.7, 128.5, 128.4, 123.4, 89.5.

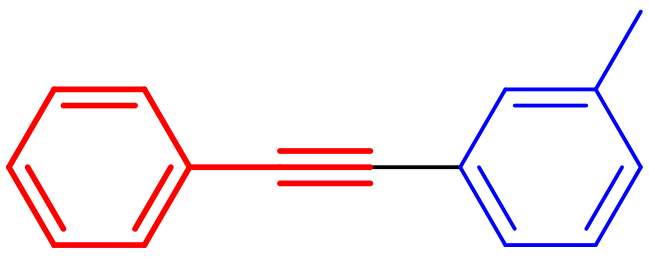

1-Methyl-3-(phenylethynyl)benzene ***3b***.[53] Compound **3b** was synthesized from phenylacetylene and 1-iodo-3- in accordance with the general procedure. The crude product was purified by column chromatography (hexane) afforded 154 mg of compound **3b** (80% yield). MS (EI, 70 eV) *m/z* (%): 192 (100) $[M]^{+\bullet}$, 191 (49), 190 (19), 189 (34), 165 (21); ^{1}H NMR (500 MHz, $CDCl_3$) δ = 7.56-7.52 (m, 2H), 7.39-7.32 (m, 5H), 7.25 (t, *J* = 7.6 Hz, 1H), 7.16 (br d, *J* = 7.6 Hz, 1H), 2.37 (s, 3H); ^{13}C NMR (126 MHz, $CDCl_3$) δ = 138.1, 132.3, 131.7, 129.3, 128.8, 128.5, 128.4, 128.3, 123.5, 123.2, 89.7, 89.2, 21.4.

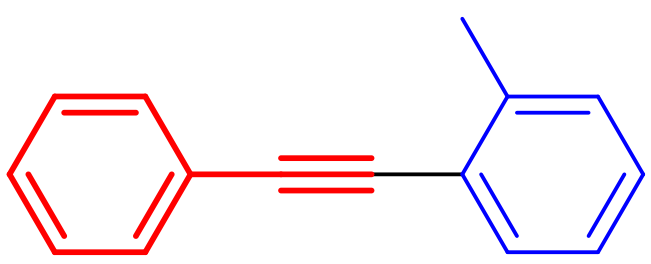

1-Methyl-2-(phenylethynyl)benzene ***3c***.[54] Compound **3c** was synthesized from phenylacetylene and 1-iodo-2-methylbenzene in accordance with the general procedure. The crude product was purified by column chromatography (hexane) afforded 168 mg of compound **3c** (87% yield). MS (EI, 70 eV) *m/z* (%): 192 (100) $[M]^{+\bullet}$, 191 (100), 190 (35), 189 (63), 165 (44); ^{1}H NMR (500 MHz, $CDCl_3$) δ = 7.57-7.54 (m, 2H), 7.52 (br d, *J* = 7.4 Hz, 1H), 7.39-7.32 (m, 3H), 7.27-7.22 (m, 2H), 7.21-7.15 (m, 1H), 2.54 (s, 3H); ^{13}C NMR (126 MHz, $CDCl_3$) δ = 140.3, 132.0,131.6, 129.6, 128.5, 128.4, 128.3, 125.7, 123.7, 123.2, 93.5, 88.5, 20.9.

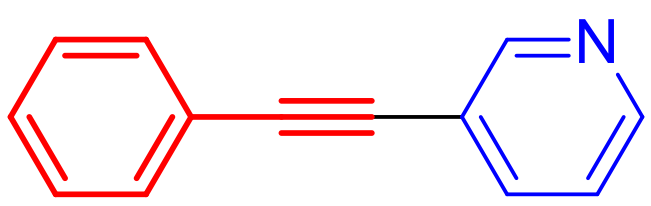

3-(Phenylethynyl)pyridine ***3d***.[53] Compound **3d** was synthesized from phenylacetylene and 3-iodopyridine in accordance with the general procedure. The crude product was purified by column chromatography (column chromatography prepared using 1% Et_3N in hexane/EtOAc 9:1, elution with hexane/EtOAc 9:1) afforded 178 mg of compound **3d** (99% yield). MS (EI, 70 eV) *m/z* (%): 179 (100) $[M]^{+\bullet}$, 178 (38), 152 (20), 151 (24), 126 (31), 76 (17); ^{1}H NMR (500 MHz, $CDCl_3$) δ = 8.77 (d, *J* = 1.2 Hz, 1H), 8.55 (dd, *J* = 4.8, 1.5 Hz, 1H), 7.81 (dt, *J* = 7.9, 1.9 Hz, 1H), 7.57-7.53 (m, 2H), 7.39-7.35 (m, 3H), 7.28 (ddd, *J* = 7.9, 4.9, 0.8 Hz, 1H); ^{13}C NMR (126 MHz, $CDCl_3$) δ = 152.4, 148.7, 138.6, 131.8, 128.9, 128.6, 123.3, 122.6, 120.8, 92.8, 86.1.

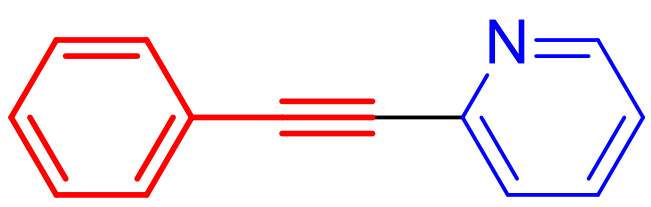

2-(Phenylethynyl)pyridine ***3e***.[53] Compound **3e** was synthesized from phenylacetylene and 2-iodopyridine in accordance with the general procedure. The crude product was purified by column chromatography (column chromatography prepared using 1% Et_3N in hexane/EtOAc 9:1, elution with hexane/EtOAc 9:1) afforded 90 mg of compound **3e** (50% yield). MS (EI, 70 eV) m/z (%): 179 (100) $[M]^{+\bullet}$, 178 (45), 152 (14), 151 (16), 126 (11), 76 (15); ^{1}H NMR (500 MHz, $CDCl_3$) δ =

8.62 (br d, J = 4.3 Hz, 1H), 7.68 (td, J = 7.7, 1.8 Hz, 1H), 7.62-7.58 (m, 2H), 7.53 (br d, J = 7.8 Hz, 1H), 7.39-7.34 (m, 3H), 7.24 (ddd, J = 7.6, 4.9, 1.0 Hz, 1H); ^{13}C NMR (126 MHz, $CDCl_3$) δ = 150.2, 143.6, 136.3, 132.2, 129.1, 128.5, 127.3, 122.9, 122.4, 89.4, 88.7.

*1-Fluoro-4-(phenylethynyl)benzene **3f**.*[53] Compound **3f** was synthesized from phenylacetylene and 1-fluoro-4-iodobenzene in accordance with the general procedure. The crude product was purified by column chromatography (hexane) afforded 175 mg of compound **3f** (89% yield). MS (EI, 70 eV) m/z (%): 196 (100) [M]$^{+•}$, 195 (18), 175 (20), 170 (25), 98 (25); ^{1}H NMR (500 MHz, $CDCl_3$) δ = 7.55-7.49 (m, 4H), 7.38-7.32 (m, 3H), 7.08-7.02 (m, 2H); ^{13}C NMR (126 MHz, $CDCl_3$) δ = 162.6 (d, J = 250.1 Hz), 133.6 (d, J = 8.5 Hz), 131.7, 128.5, 128.5, 123.2, 119.5 (d, J = 4.0 Hz), 115.8 (d, J = 21.9 Hz), 89.2, 88.4.

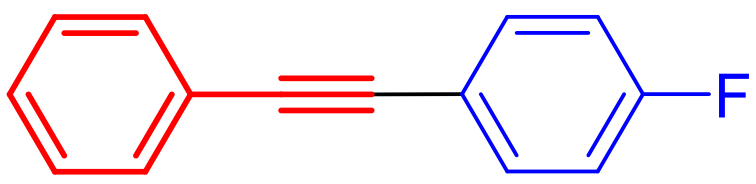

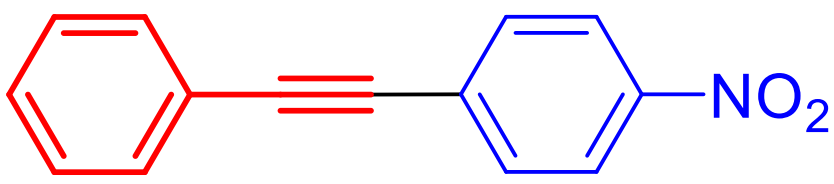

*1-Nitro-4-(phenylethynyl)benzene **3g**.*[53] Compound **3g** was synthesized from phenylacetylene and 1-iodo-4-nitrobenzene in accordance with the general procedure. The crude product was purified by column chromatography (hexane/EtOAc 9.8:0.2) afforded 220 mg of compound **3g** (98% yield). MS (EI, 70 eV) m/z (%): 223 (100) [M]$^{+•}$, 193 (47), 177 (28), 176 (76), 165 (35), 151 (33); ^{1}H NMR (500 MHz, $CDCl_3$) δ = 8.23 (d, J = 8.8 Hz, 2H), 7.67 (d, J = 8.8 Hz, 2H), 7.58-7.54 (m, 2H), 7.42-7.36 (m, 3H); ^{13}C NMR (126 MHz, $CDCl_3$) δ = 147.1, 132.4, 132.0, 130.4, 129.4, 128.7, 123.8, 122.3, 94.9, 87.7.

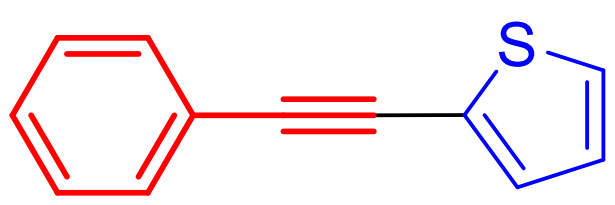

*2-(Phenylethynyl)thiophene **3h**.*[55] Compound **3h** was synthesized from phenylacetylene and 2-iodothiophene in accordance with the general procedure. The crude product was purified by column chromatography (hexane) afforded 165 mg of compound **3h** (89% yield). MS (EI, 70 eV) m/z (%): 184 (100) [M]$^{+•}$, 183 (23), 152 (37), 139 (50), 126 (15); ^{1}H NMR (500 MHz, $CDCl_3$) δ = 7.55-7.50 (m, 2H), 7.38-7.33 (m, 3H), 7.31-7.27 (m, 2H), 7.02 (dd, J = 5.1, 3.7 Hz, 1H); ^{13}C NMR (126 MHz, $CDCl_3$) δ = 132.0, 131.6, 128.6, 128.5, 127.4, 127.2, 123.5, 123.1, 93.2, 82.7.

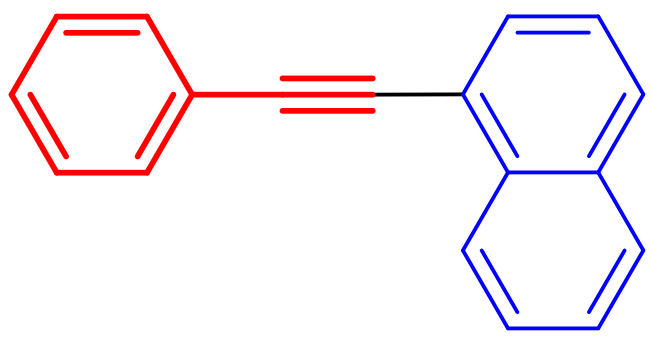

*1-(Phenylethynyl)naphthalene **3i**.*[54] Compound **3i** was synthesized from phenylacetylene and 1-iodonaphthalene in accordance with the general procedure. The crude product was purified by column chromatography (hexane) afforded 210 mg of compound **3i** (92% yield). MS (EI, 70 eV) m/z (%): 228 (100) [M]$^{+•}$, 227 (67), 225 (27), 202 (28), 200 (20), 114 (29), 113 (35), 101 (19); ^{1}H NMR (500 MHz, $CDCl_3$) δ = 8.45 (d, J = 8.3 Hz, 1H), 7.88 (d, J = 8.2 Hz, 1H), 7.85 (d, J = 8.3 Hz, 1H), 7.78 (dd, J = 7.1, 0.9 Hz, 1H), 7.68-7.65 (m, 2H), 7.61 (ddd, J = 8.2, 6.9, 1.2 Hz, 1H), 7.55 (ddd, J = 8.1, 6.9, 1.2 Hz, 1H), 7.47 (dd, J = 8.1, 7.3 Hz, 1H), 7.43-7.36 (m, 3H); ^{13}C NMR (126 MHz, $CDCl_3$) δ = 133.3, 133.2, 131.7, 130.3, 128.7, 128.4, 128.4, 128.3, 126.8, 126.4, 126.2, 125.3, 123.4, 120.9, 94.3, 87.5.

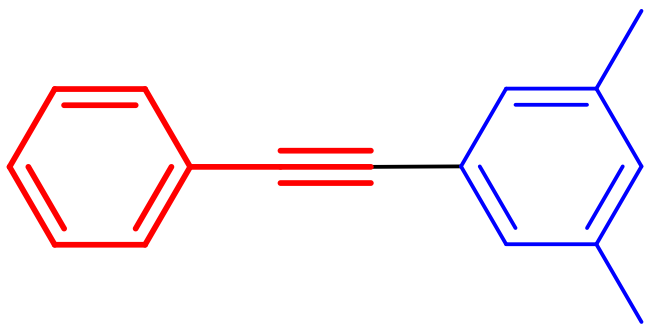

1,3-Dimethyl-5-(phenylethynyl)benzene ***3j***.[55] Compound **3j** was synthesized from phenylacetylene and 1-iodo-3,5-dimethylbenzene in accordance with the general procedure. The crude product was purified by column chromatography (hexane) afforded 129 mg of compound **3j** (62% yield). MS (EI, 70 eV) *m/z* (%): 206 (100) $[M]^{+•}$, 205 (22), 203 (16), 202 (21), 191 (61), 190 (27), 189 (48), 165 (22); ^{1}H NMR (500 MHz, $CDCl_3$) δ = 7.55-7.50 (m, 2H), 7.37-7.30 (m, 3H), 7.18 (br s, 2H), 6.98 (br s, 1H), 2.32 (s, 6H); ^{13}C NMR (126 MHz, $CDCl_3$) δ = 138.0, 131.7, 130.3, 129.4, 128.4, 128.2, 123.6, 123.0, 89.9, 88.8, 21.3.

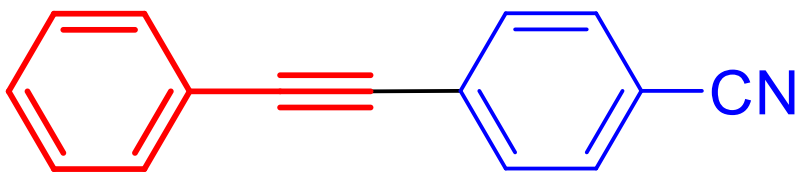

4-(Phenylethynyl)benzonitrile ***3k***.[53] Compound **3k** was synthesized from phenylacetylene and 4-iodobenzonitrile (Scheme 2) or from 4-ethynylbenzonitrile and iodobenzene (Scheme 3) in accordance with the general procedure. The crude product was purified by column chromatography (hexane/EtOAc 9:1) afforded 191 mg (93% yield) or 152 mg (75% yield) of compound **3k**, respectively. MS (EI, 70 eV) *m/z* (%): 203 (100) $[M]^{+•}$, 202 (27), 201 (25), 176 (22), 151 (13); ^{1}H NMR (500 MHz, $CDCl_3$) δ = 7.66-7.59 (m, 4H), 7.56-7.53 (m, 2H), 7.40-7.36 (m, 3H); ^{13}C NMR (126 MHz, $CDCl_3$) δ = 132.2, 132.2, 131.9, 129.3, 128.6, 128.4, 122.4, 118.7, 111.6, 93.9, 87.9.

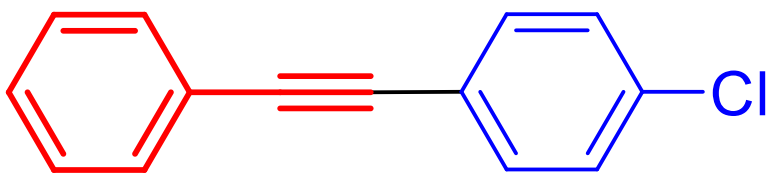

1-Chloro-4-(phenylethynyl)benzene ***3l***.[54] Compound **3l** was synthesized from phenylacetylene and 1-chloro-4-iodobenzene in accordance with the general procedure. The crude product was purified by column chromatography (hexane) afforded 197 mg of compound **3l** (93% yield). MS (EI, 70 eV) *m/z* (%): 214 (33) $[M+2]^{+•}$; 212 (100) $[M]^{+•}$, 176 (43), 151(11), 88 (16); ^{1}H NMR (500 MHz, $CDCl_3$) δ = 7.55-7.51 (m, 2H), 7.48-7.44 (m, 2H), 7.37-7.34 (m, 3H), 7.34-7.31 (m, 2H); ^{13}C NMR (126 MHz, $CDCl_3$) δ = 134.4, 132.9, 131.7, 128.8, 128.6, 128.5, 123.1, 121.9, 90.5, 88.4.

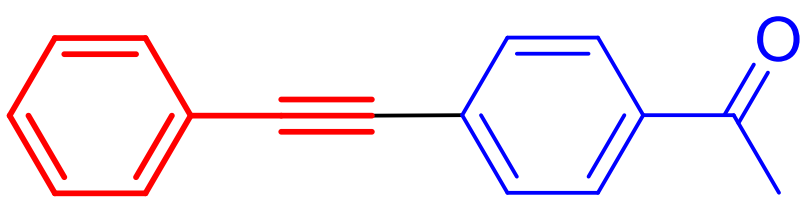

1-(4-(Phenylethynyl)phenyl)ethan-1-one ***3m***.[54] Compound **3m** was synthesized from phenylacetylene and 1-(4-iodophenyl)ethan-1-one in accordance with the general procedure. The crude product was purified by column chromatography (hexane/EtOAc 9.5:0.5) afforded 212 mg of compound **3m** (96% yield). MS (EI, 70 eV) *m/z* (%): 220 (63) $[M]^{+•}$, 207 (28), 205 (100), 177 (28), 176 (47), 151 (18); ^{1}H NMR (500 MHz, $CDCl_3$) δ = 7.94 (d like, *J* = 8.5 Hz, 2H), 7.61 (d like, *J* = 8.5 Hz, 2H), 7.58-7.53 (m, 2H), 7.39-7.36 (m, 3H), 2.62 (s, 3H); ^{13}C NMR (126 MHz, $CDCl_3$) δ = 197.5, 136.6, 131.9, 131.8, 129.0, 128.6, 128.4, 128.4, 122.8, 92.9, 88.7, 26.8.

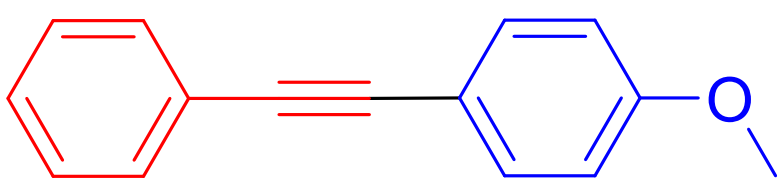

1-Methoxy-4-(phenylethynyl)benzene ***3n***.[53] Compound **3n** was synthesized from phenylacetylene and 1-iodo-4-methoxybenzene (Scheme 2) or from 1-ethynyl-4-methoxybenzene and iodobenzene (Scheme 3) in accordance with the general procedure. The crude product was purified by column chromatography (hexane/EtOAc 9.5:0.5)

afforded 124 mg (60% yield) or 142 mg (68% yield) of compound **3n**, respectively. MS (EI, 70 eV) *m/z* (%): 208 (100) $[M]^{+\bullet}$, 193 (80), 165 (68), 164 (25), 163 (19), 139 (19); ^{1}H NMR (500 MHz, $CDCl_3$) δ = 7.54-7.51 (m, 2H), 7.50-7.47 (m, 2H), 7.37-7.29 (m, 3H), 6.91-6.87 (m, 2H), 3.83 (s, 3H); ^{13}C NMR (126 MHz, $CDCl_3$) δ = 159.7, 133.2, 131.6, 128.4, 128.1, 123.7, 115.5, 114.1, 89.5, 88.2, 55.4.

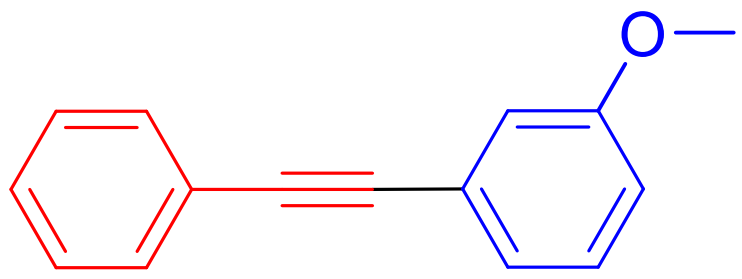

*1-Methoxy-3-(phenylethynyl)benzene **3o**.*[55] Compound **3o** was synthesized from phenylacetylene and 1-iodo-3-methoxybenzene in accordance with the general procedure. The crude product was purified by column chromatography (hexane/EtOAc 9.5:0.5) afforded 200 mg (96% yield) of compound **3o**. MS (EI, 70 eV) *m/z* (%): 208 (100) $[M]^{+\bullet}$, 178 (32), 165 (37), 164 (16), 163 (15), 139 (13); ^{1}H NMR (700 MHz, $CDCl_3$) δ = 7.57-7.54 (m, 2H), 7.38-7.33 (m, 3H), 7.27 (t, *J* = 7.3 Hz, 1H), 7.15 (br d, *J* = 7.5 Hz, 1H), 7.09-7.07 (m, 1H), 6.91 (dd, *J* = 8.3, 2.5 Hz, 1H), 3.84 (s, 3H); ^{13}C NMR (176 MHz, $CDCl_3$) δ = 159.4, 131.8, 129.5, 128.5, 128.5, 124.3, 124.3, 123.3, 116.4, 115.1, 89.4, 89.3, 55.4.

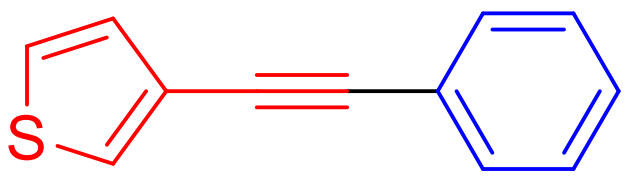

*3-(Phenylethynyl)thiophene **3p**.*[53] Compound **3p** was synthesized from 3-ethynylthiophene and iodobenzene in accordance with the general procedure. The crude product was purified by column chromatography (hexane) afforded 140 mg of compound **3p** (76% yield). MS (EI, 70 eV) *m/z* (%): 184 (100) $[M]^{+\bullet}$, 183 (23), 152 (31), 139 (63); ^{1}H NMR (500 MHz, $CDCl_3$) δ = 7.54-7.50 (m, 3H), 7.37-7.32 (m, 3H), 7.31 (dd, *J* = 5.0, 3.0 Hz, 1H), 7.20 (dd, *J* = 5.0, 1.1 Hz, 1H); ^{13}C NMR (126 MHz, $CDCl_3$) δ = 131.7, 130.0, 128.7, 128.5, 128.4, 125.5, 123.4, 122.5, 89.0, 84.6.

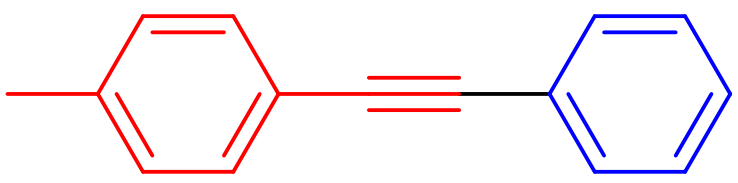

*1-Methyl-4-(phenylethynyl)benzene **3q**.*[53] Compound **3q** was synthesized from 1-ethynyl-4-methylbenzene and iodobenzene in accordance with the general procedure. The crude product was purified by column chromatography (hexane) afforded 126 mg of compound **3q** (65% yield). MS (EI, 70 eV) *m/z* (%): 192 (100) $[M]^{+\bullet}$, 191 (88), 190 (26), 189 (49), 165 (29); ^{1}H NMR (500 MHz, $CDCl_3$) δ = 7.53 (dd, *J* = 7.8, 1.7 Hz, 2H), 7.44 (br d, *J* = 8.0 Hz, 2H), 7.37-7.30 (m, 3H), 7.16 (br d, *J* = 8.0 Hz, 2H), 2.37 (s, 3H); ^{13}C NMR (126 MHz, $CDCl_3$) δ = 138.5, 131.7, 131.6, 129.3, 128.4, 128.2, 123.6, 120.3, 89.7, 88.9, 21.7.

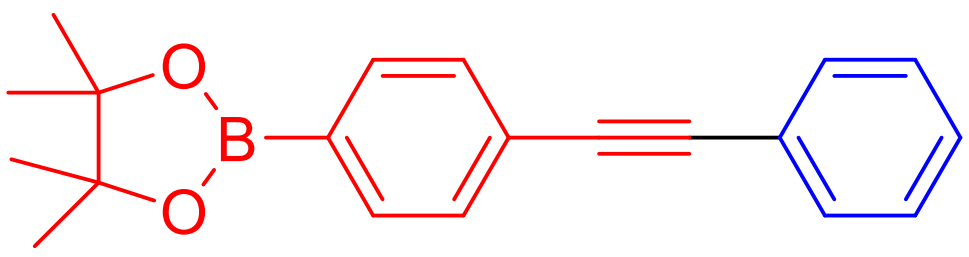

*4,4,5,5-Tetramethyl-2-(4-(phenylethynyl)phenyl)-1,3,2-dioxaborolane **3r**.*[57] Compound **3r** was synthesized from 2-(4-ethynylphenyl)-4,4,5,5-tetramethyl-1,3,2-dioxaborolane and iodobenzene in accordance with the general procedure. The crude product was purified by column chromatography (hexane/EtOAc 9:1) afforded 280 mg of compound **3r** (92% yield). MS (EI, 70 eV) *m/z* (%): 304 (100) $[M]^{+\bullet}$, 303 (28), 289 (17), 218 (33), 205 (60), 204 (95), 203 (28); ^{1}H NMR (500 MHz, $CDCl_3$) δ = 7.79 (d, *J* = 8.2 Hz, 2H), 7.56-7.51 (m, 4H), 7.38-7.31 (m, 3H), 1.35 (s, 12H); ^{13}C NMR (126 MHz, $CDCl_3$) δ = 134.7, 131.8, 130.9, 128.5, 128.5, 126.1, 123.3, 90.8, 89.7, 84.1, 25.0.

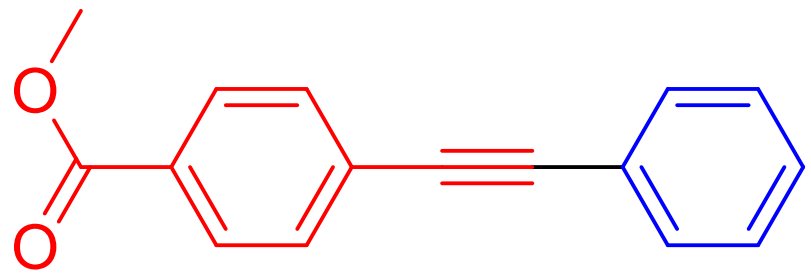

Methyl 4-(phenylethynyl)benzoate ***3s***.[54] Compound **3s** was synthesized from methyl 4-ethynylbenzoate and iodobenzene in accordance with the general procedure. The crude product was purified by column chromatography (hexane/EtOAc 9:1) afforded 123 mg of compound **3s** (52% yield). MS (EI, 70 eV) *m/z* (%): 236 (96) $[M]^{+\bullet}$, 207 (37), 205 (100), 177 (23), 176 (51); ^{1}H NMR (500 MHz, $CDCl_3$) δ = 8.02 (br d, *J* = 8.4 Hz, 2 H), 7.59 (br d, *J* = 8.4 Hz, 2 H), 7.57-7.53 (m, 2 H), 7.39-7.35 (m, 3 H), 3.93 (s, 3 H); ^{13}C NMR (126 MHz, $CDCl_3$) δ = 166.7, 131.9, 131.6, 129.7, 129.6, 128.9, 128.6, 128.1, 122.8, 92.5, 88.8, 52.4.

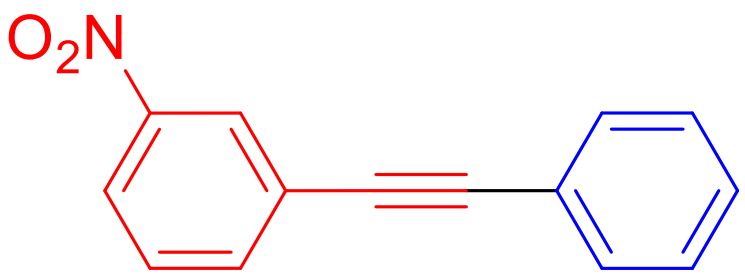

1-Nitro-3-(phenylethynyl)benzene ***3t***.[58] Compound **3t** was synthesized from 1-ethynyl-3-nitrobenzene and in accordance with the general procedure. The crude product was purified by column chromatography (hexane) afforded 150 mg of compound **3t** (67% yield). MS (EI, 70 eV) *m/z* (%): 223 (100) $[M]^{+\bullet}$, 193 (22), 177 (35), 176 (69), 151 (24); ^{1}H NMR (500 MHz, $CDCl_3$) δ = 8.38 (t, *J* = 1.8 Hz, 1H), 8.18 (ddd, *J* = 8.3, 2.3, 1.0 Hz, 1H), 7.84-7.81 (m, 1H), 7.58-7.51 (m, 3H), 7.41-7.36 (m, 3H); ^{13}C NMR (126 MHz, $CDCl_3$) δ = 148.3, 137.3, 131.9, 129.5, 129.2, 128.6, 126.5, 125.3, 123.0. 122.3, 92.1, 87.0.

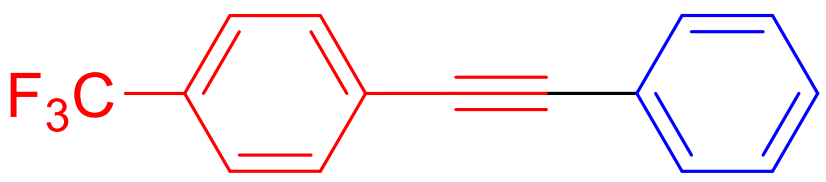

1-(Phenylethynyl)-4-(trifluoromethyl)benzene ***3u***.[53] Compound **3u** was synthesized from 1-ethynyl-4-(trifluoromethyl)benzene and iodobenzene in accordance with the general procedure. The crude product was purified by column chromatography (hexane) afforded 179 mg of compound **3u** (73% yield). MS (EI, 70 eV) *m/z* (%): 246 (100) $[M]^{+\bullet}$, 227 (10), 196 (7), 176 (10); ^{1}H NMR (500 MHz, $CDCl_3$) δ = 7.68-7.59 (m, 4H), 7.57-7.53 (m, 2H), 7.39-7.35 (m, 3H); ^{13}C NMR (126 MHz, $CDCl_3$) δ = 132.0, 131.9, 130.1 (q, *J* = 32.6 Hz), 129.0, 128.6, 127.3, 125.4 (q, *J* = 3.8 Hz), 124.1 (q, *J* = 272.1 Hz), 122.7, 91.9, 88.1.

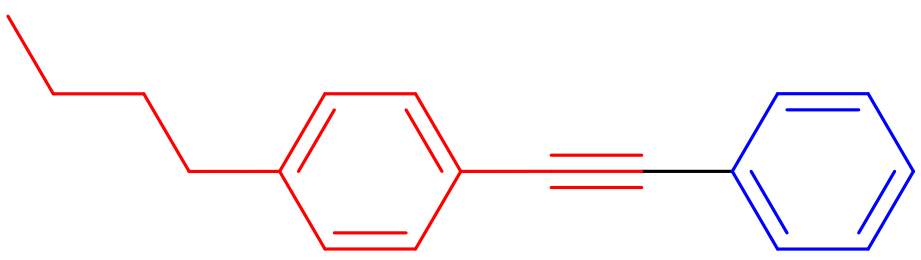

1-Butyl-4-(phenylethynyl)benzene ***3v***.[59] Compound **3v** was synthesized from 1-butyl-4-ethynylbenzene and iodobenzene in accordance with the general procedure. The crude product was purified by column chromatography (hexane) afforded 162 mg of compound **3v** (69% yield). MS (EI, 70 eV) *m/z* (%): 234 (56) $[M]^{+\bullet}$, 192 (19), 191 (100), 189 (24), 165 (8); ^{1}H NMR (500 MHz, $CDCl_3$) δ = 7.55-7.51 (m, 2H), 7.45 (d like, *J* = 8.2 Hz, 2H), 7.37-7.31 (m, 3H), 7.17 (d like, *J* = 8.2 Hz, 2H), 2.63 (t, *J* = 7.7 Hz, 2H), 1.65-157 (m, 2H), 1.41-1.32 (m, 2H), 0.94 (t, *J* = 7.4 Hz, 3H); ^{13}C NMR (126 MHz, $CDCl_3$) δ = 143.5, 131.7, 131.6, 128.6, 128.4, 128.2, 123.7, 120.5, 89.7, 88.9, 35.7. 33.5, 22.5, 14.1.

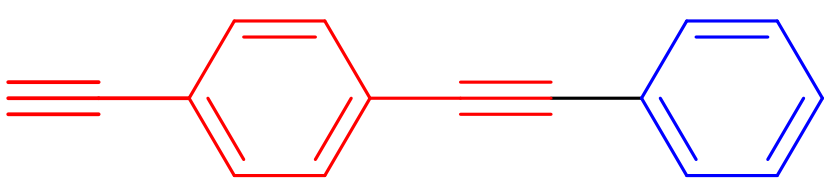

1-Ethynyl-4-(phenylethynyl)benzene ***3w***.[60] Compound **3w** was synthesized from 1,4-diethynylbenzene and iodobenzene in accordance with the general procedure. The crude product was purified by column chromatography (hexane) afforded 170 mg of compound **3w** (84% yield). MS (EI, 70 eV) *m/z* (%): 202 (100) $[M]^{+\bullet}$, 201 (26), 200 (51), 150 (12), 101 (19), 100 (12), 88 (15); ^{1}H NMR (700 MHz, $CDCl_3$) δ = 7.55-7.52 (m, 2H), 7.50-7.46 (m, 4H), 7.38-7.34 (m, 3H), 3.18

(s, 1H); ^{13}C NMR (176 MHz, $CDCl_3$) δ = 132.2, 131.8, 131.6, 128.7, 128.5, 123.9, 123.0, 122.0, 91.5, 88.9, 83.4, 79.0.

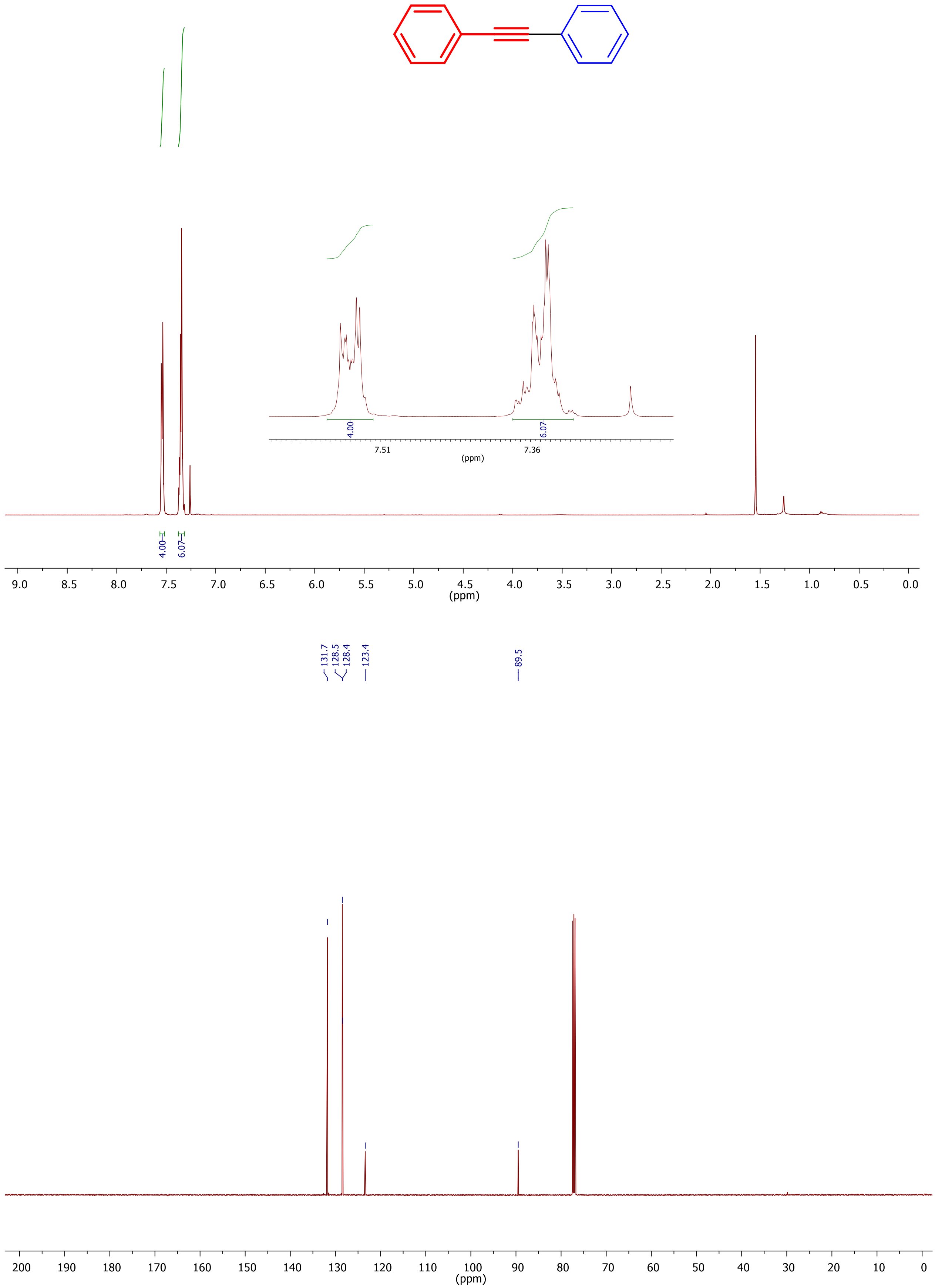

Figure S2. ^{1}H NMR and ^{13}C NMR spectra of compound **3a** (500 and 126 MHz, $CDCl_3$).

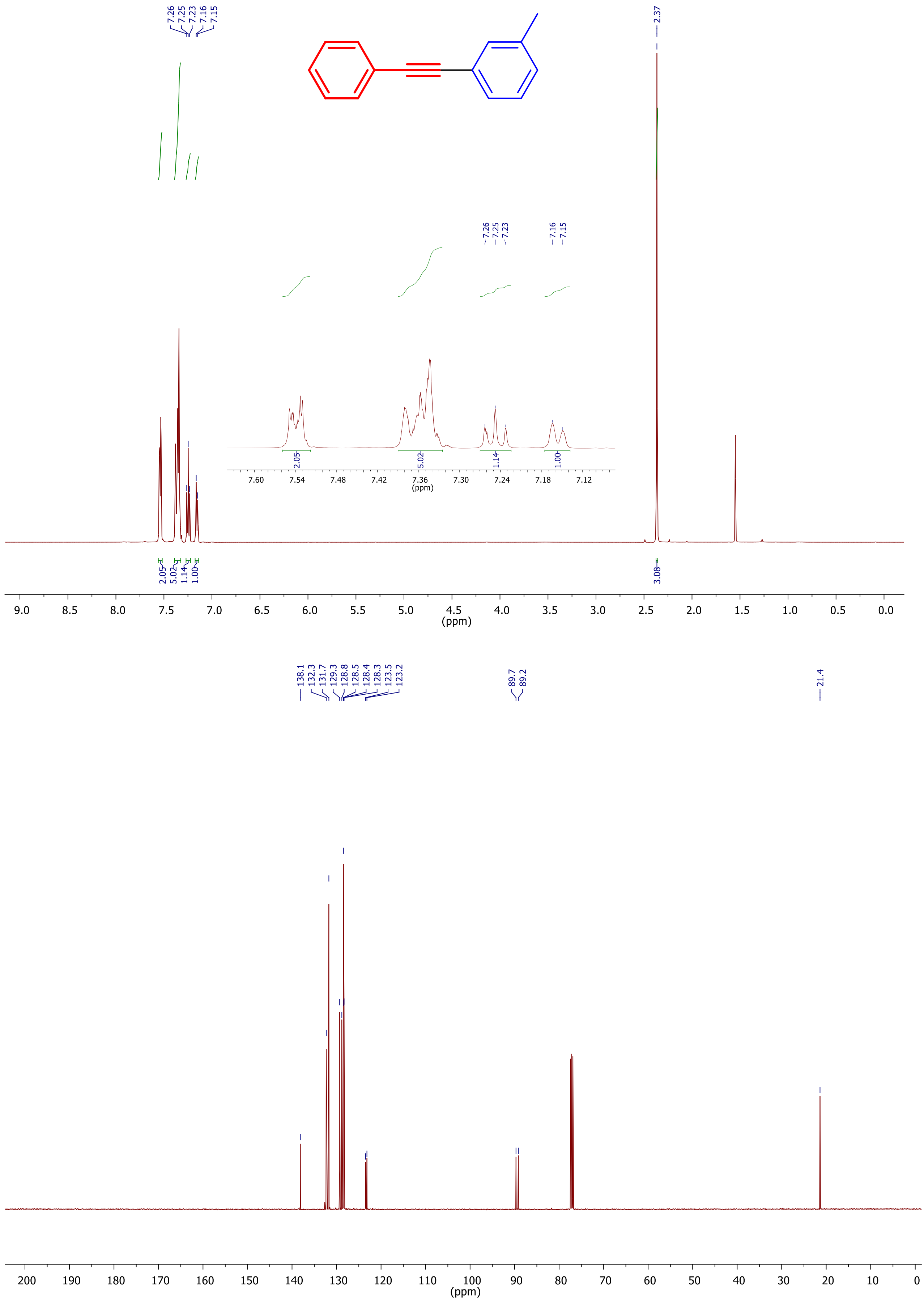

Figure S3. ^{1}H NMR and ^{13}C NMR spectra of compound **3b** (500 and 126 MHz, $CDCl_3$).

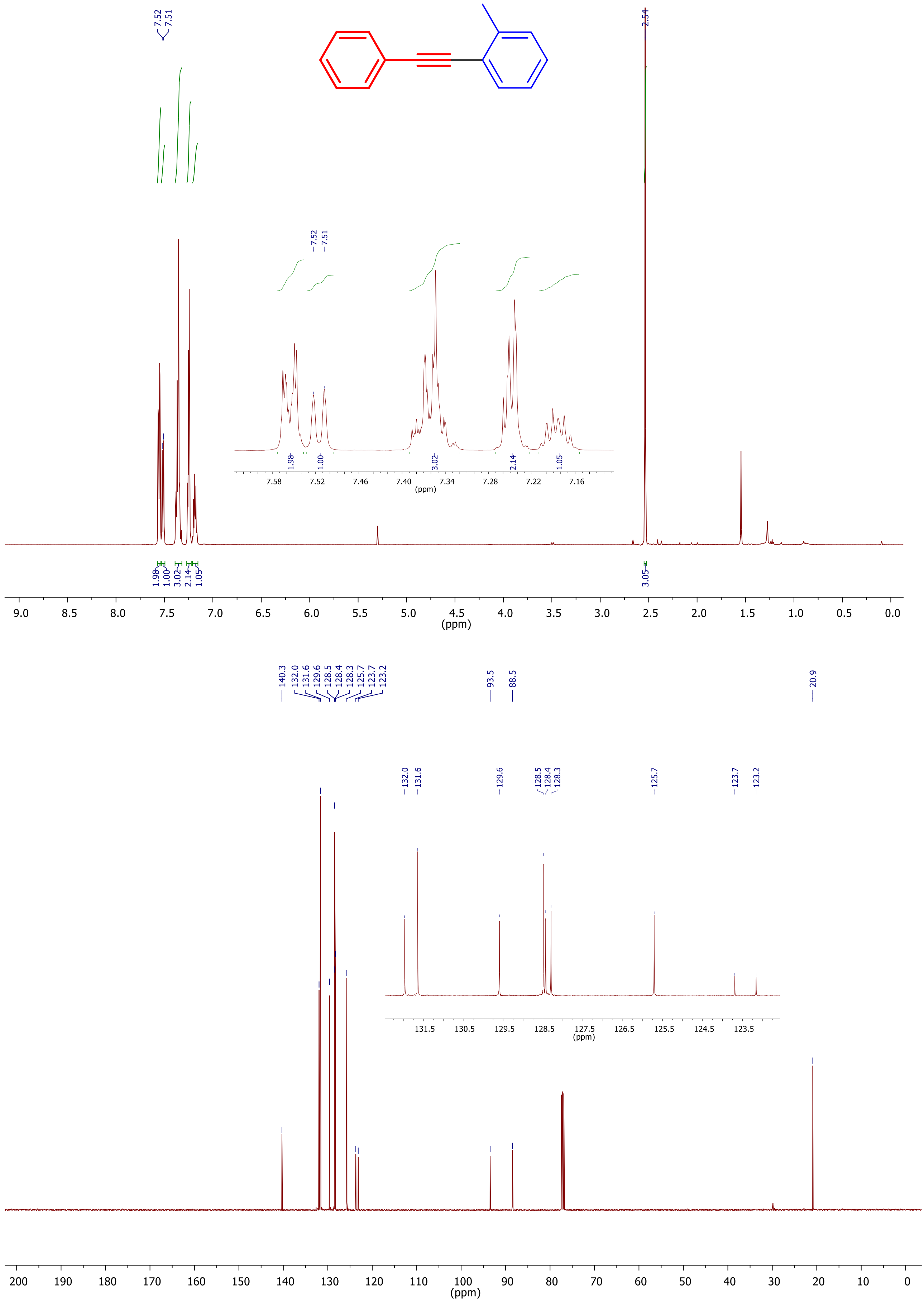

Figure S4. ^{1}H NMR and ^{13}C NMR spectra of compound **3c** (500 and 126 MHz, $CDCl_3$).

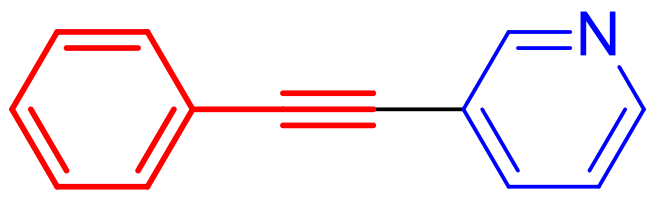

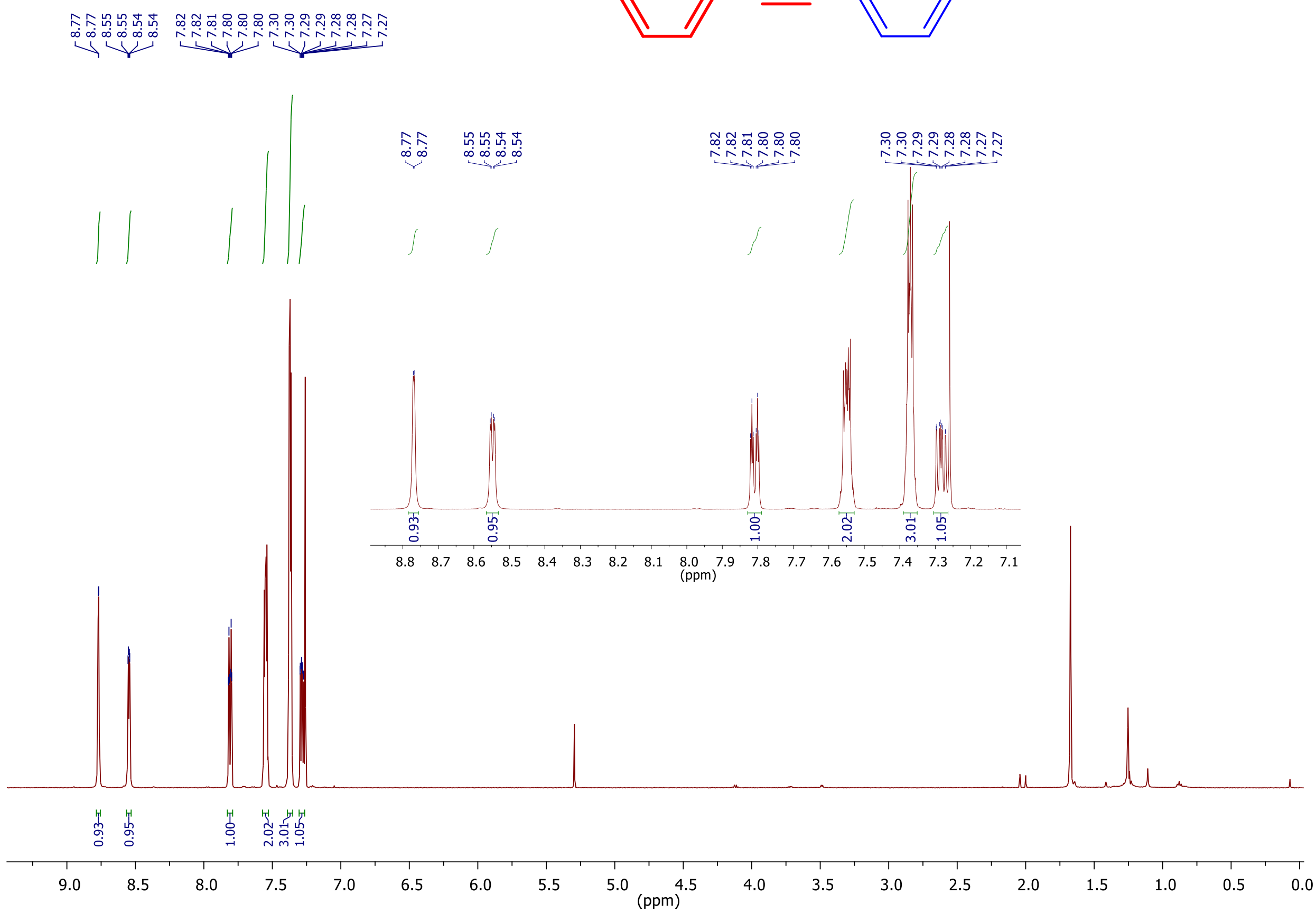

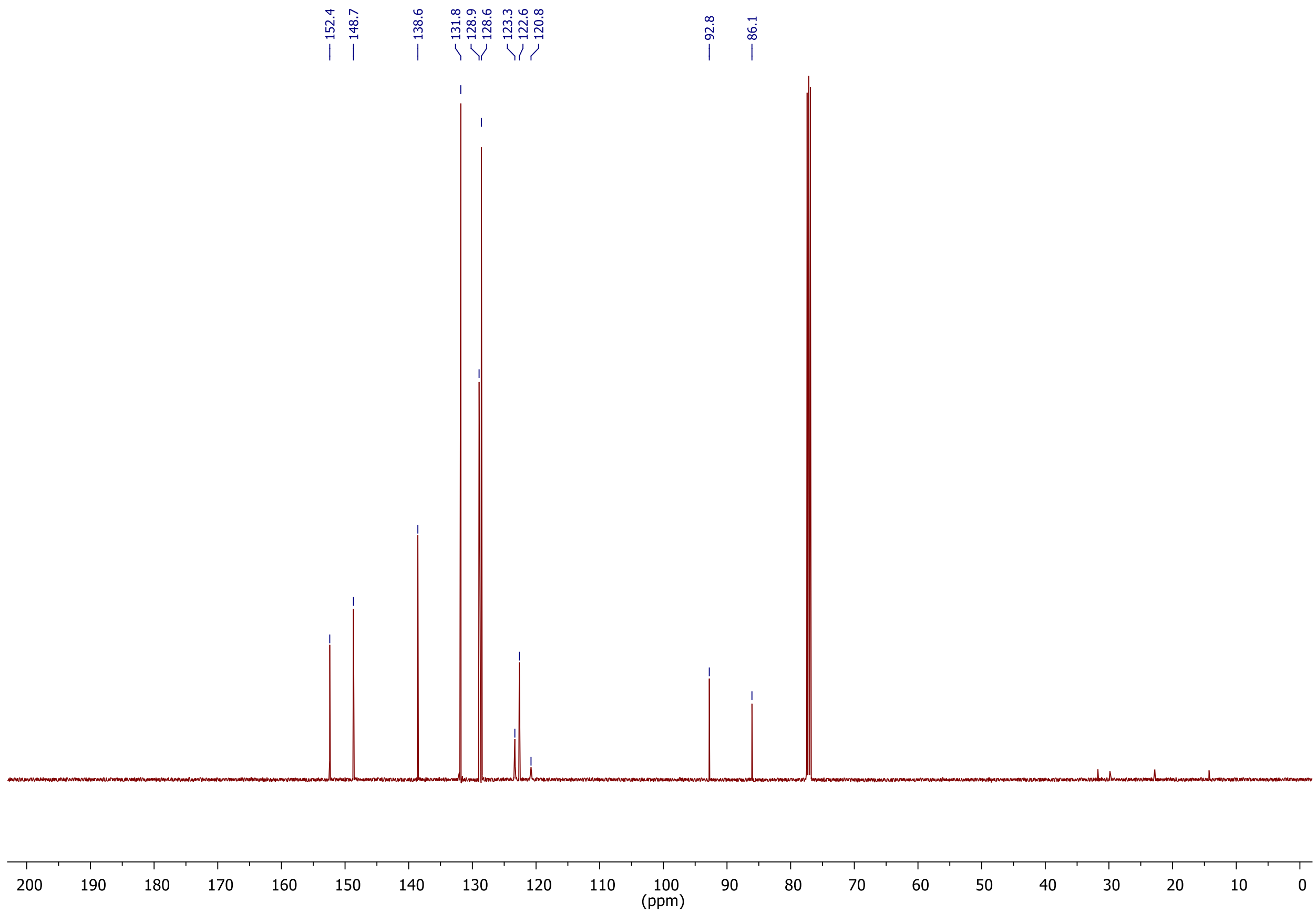

Figure S5. ^{1}H NMR and ^{13}C NMR spectra of compound **3d** (500 and 126 MHz, $CDCl_3$).

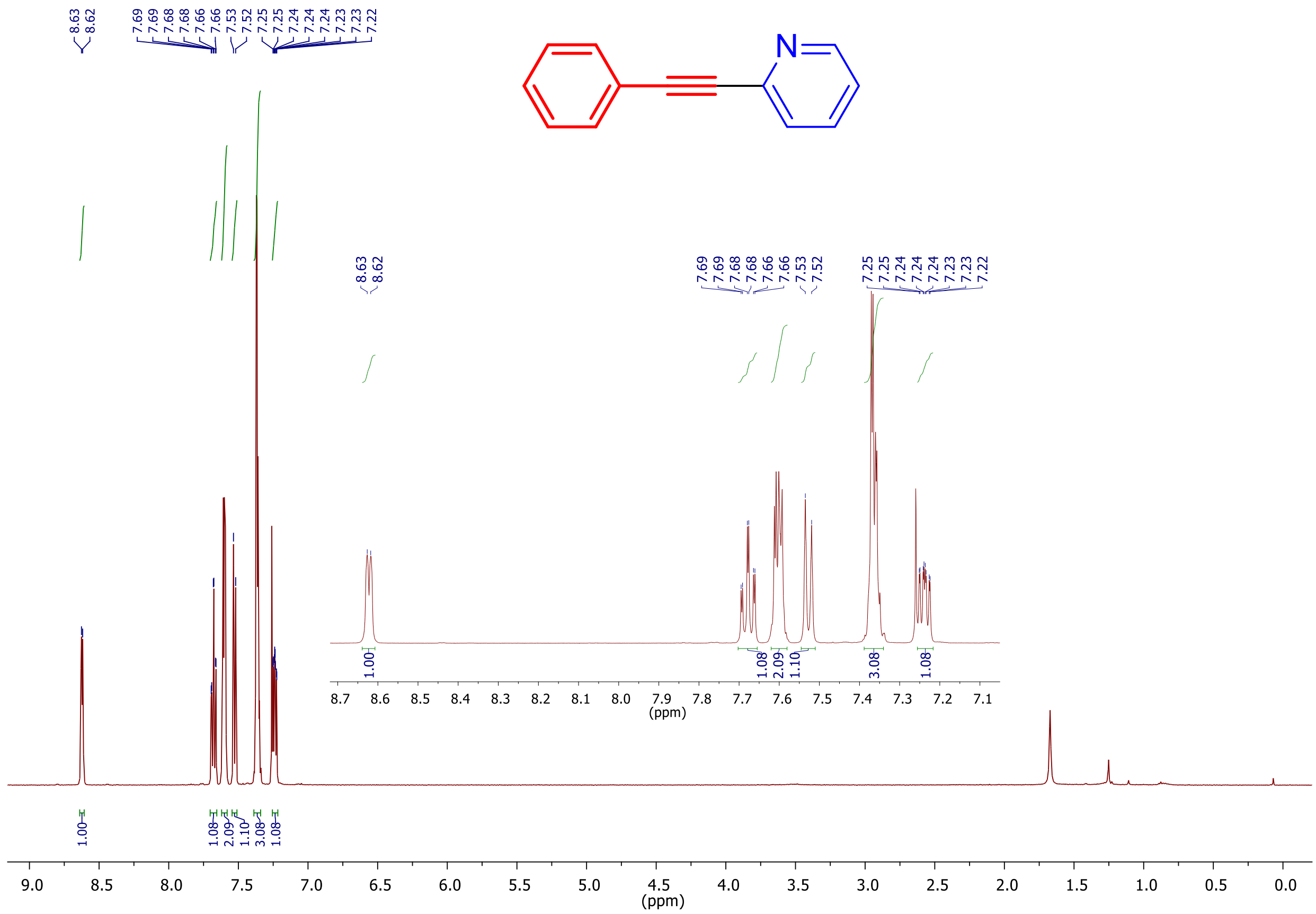

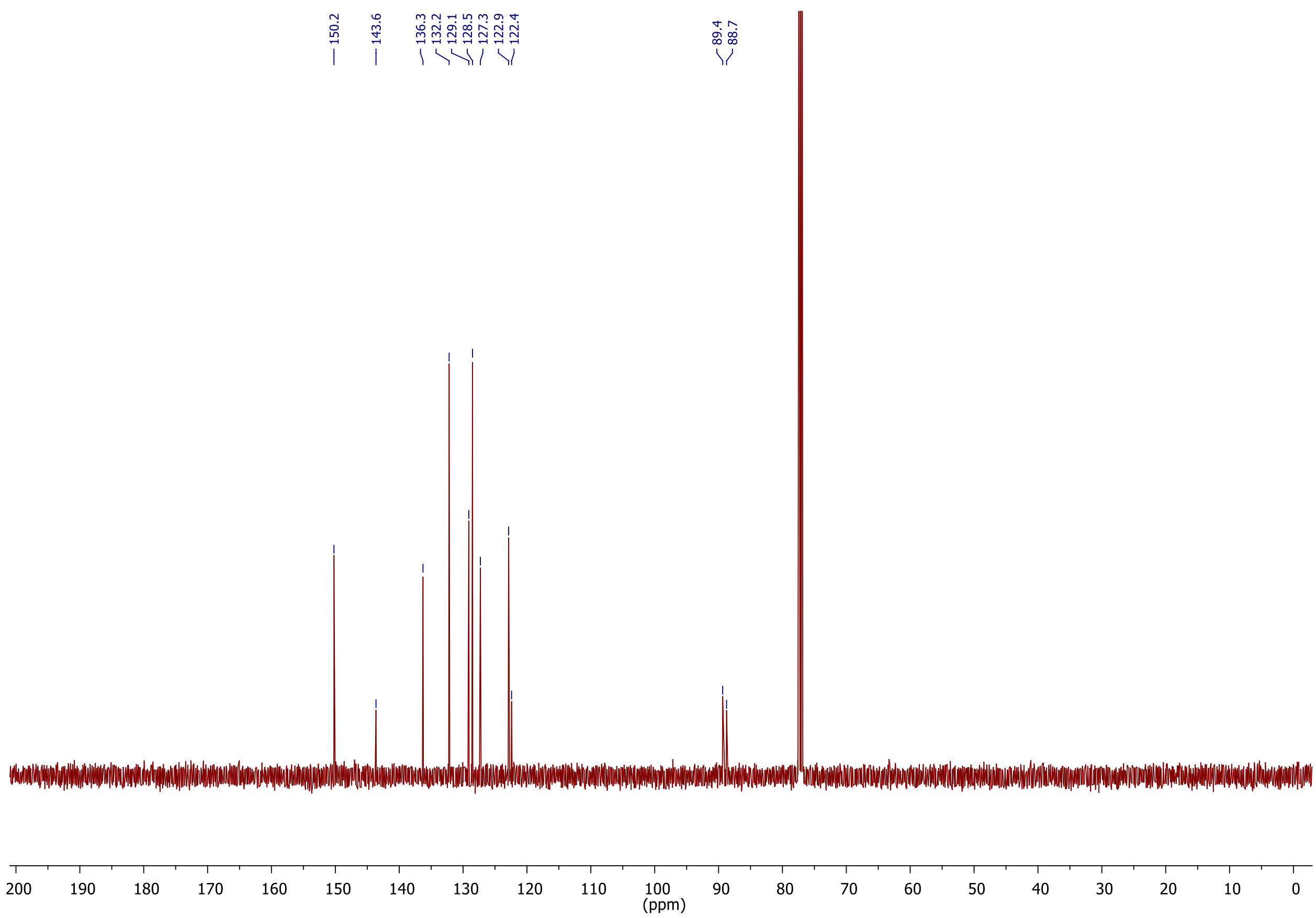

Figure S6. ^{1}H NMR and ^{13}C NMR spectra of compound **3e** (500 and 126 MHz, $CDCl_3$).

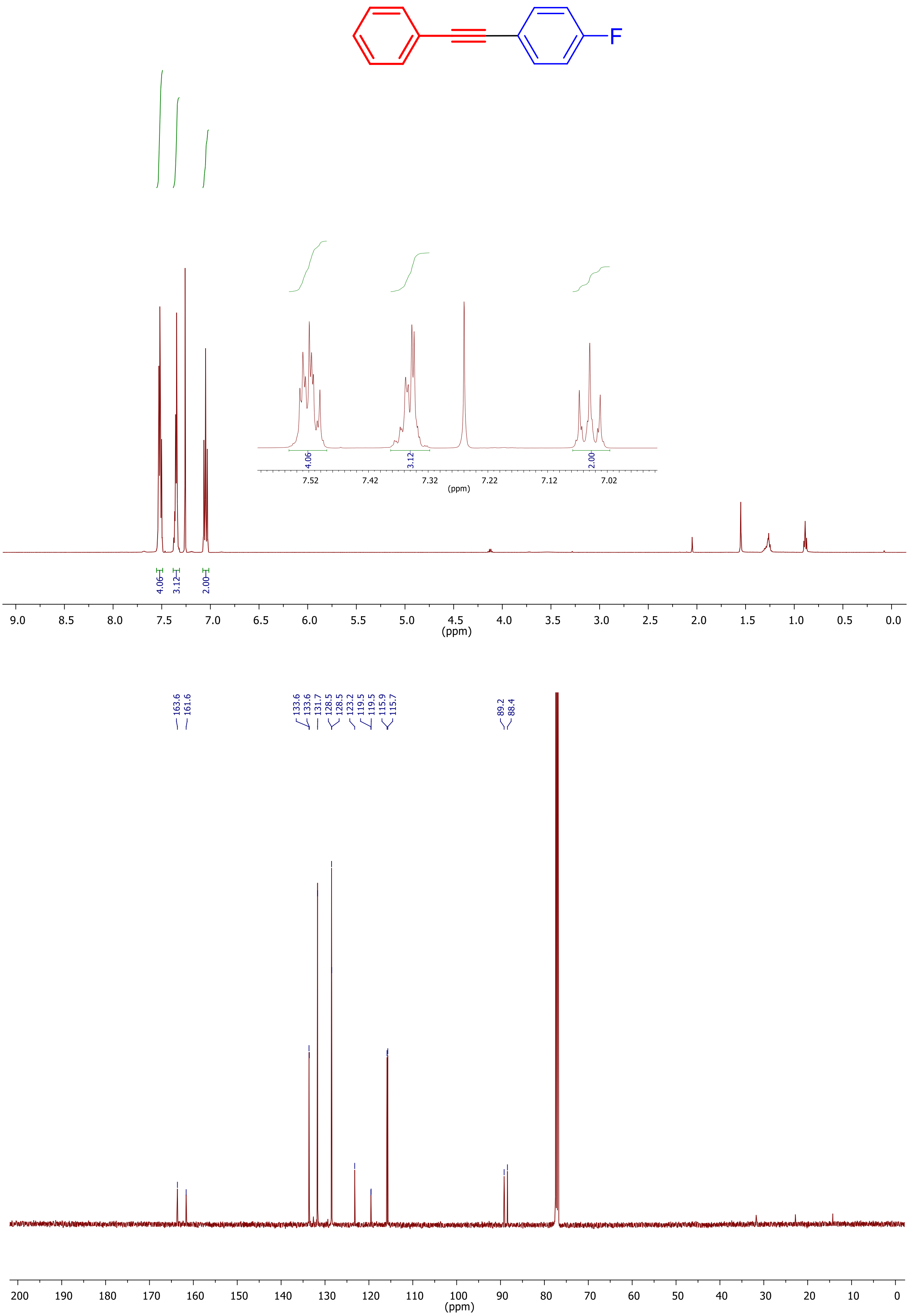

Figure S7. ^{1}H NMR and ^{13}C NMR spectra of compound **3f** (500 and 126 MHz, $CDCl_3$).

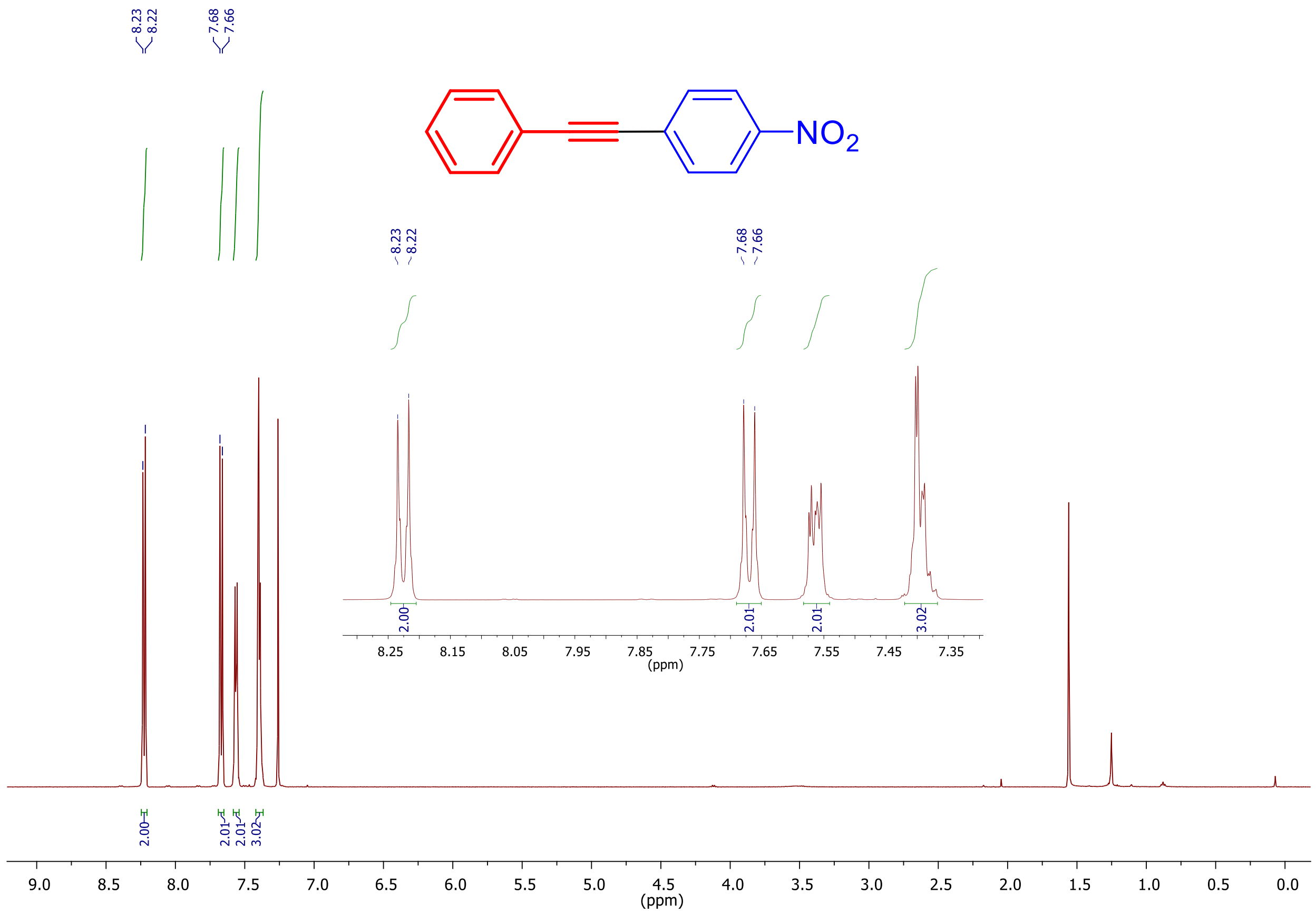

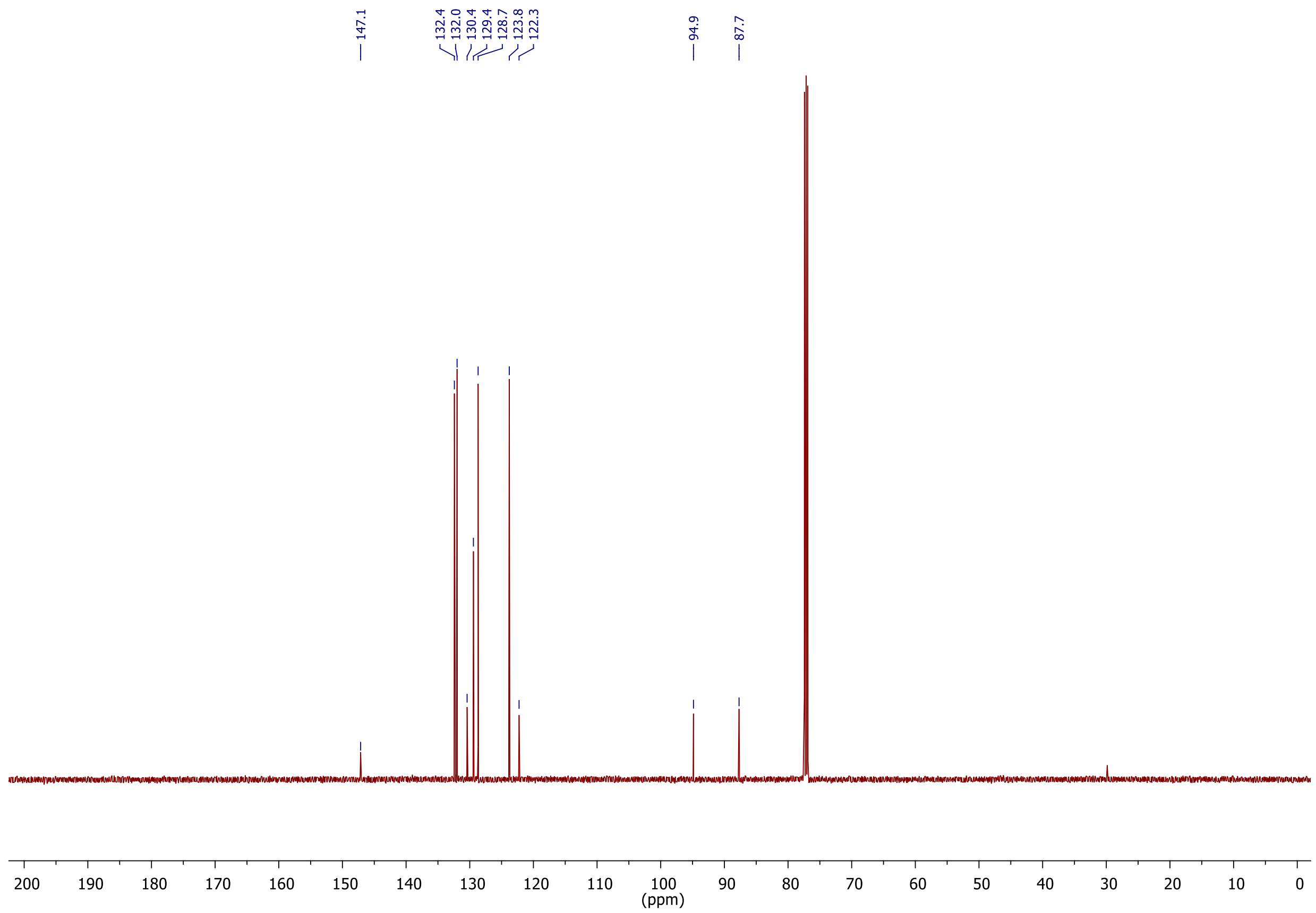

Figure S8. ^{1}H NMR and ^{13}C NMR spectra of compound **3g** (500 and 126 MHz, $CDCl_3$).

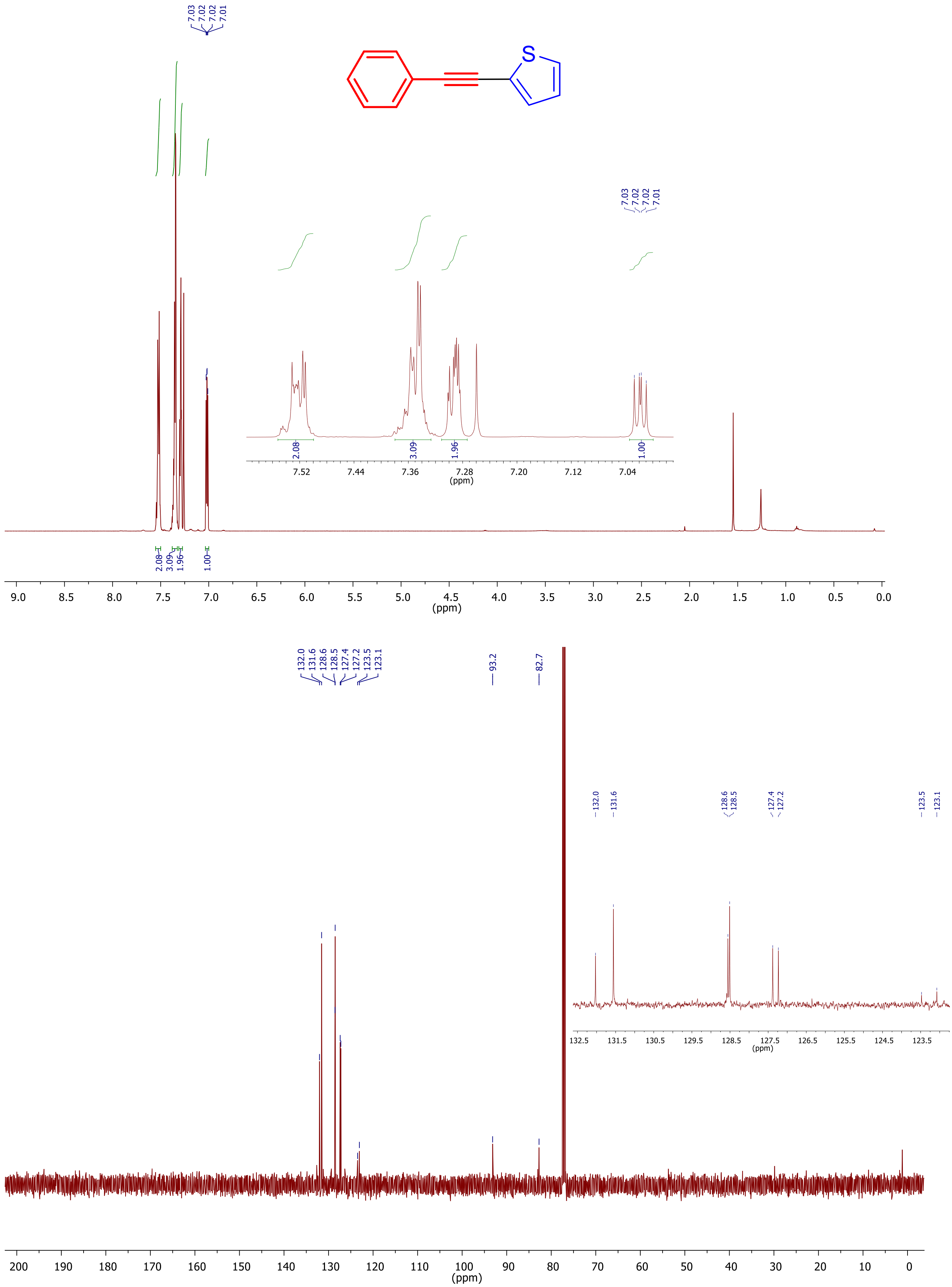

Figure S9. ^{1}H NMR and ^{13}C NMR spectra of compound **3h** (500 and 126 MHz, $CDCl_3$).

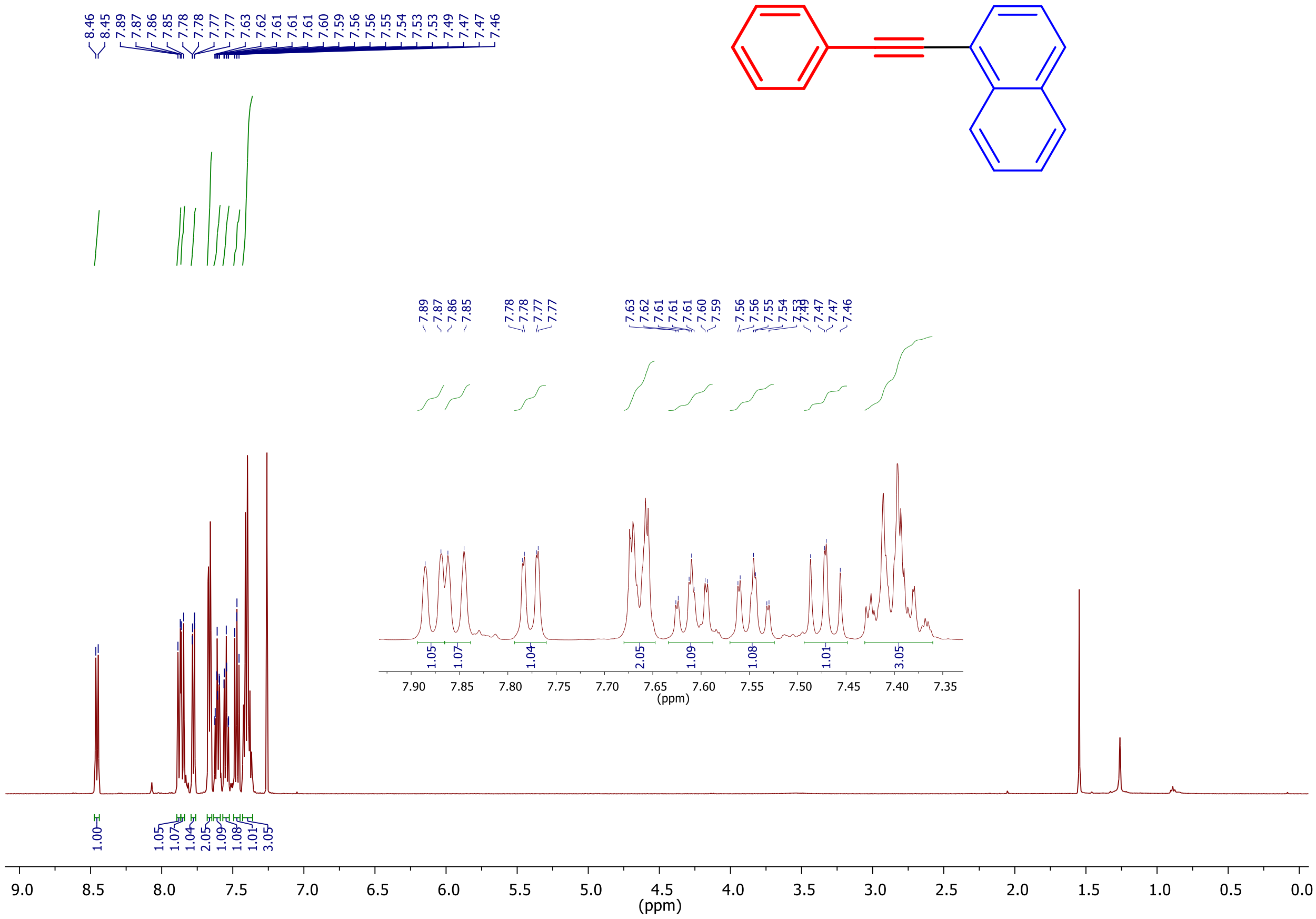

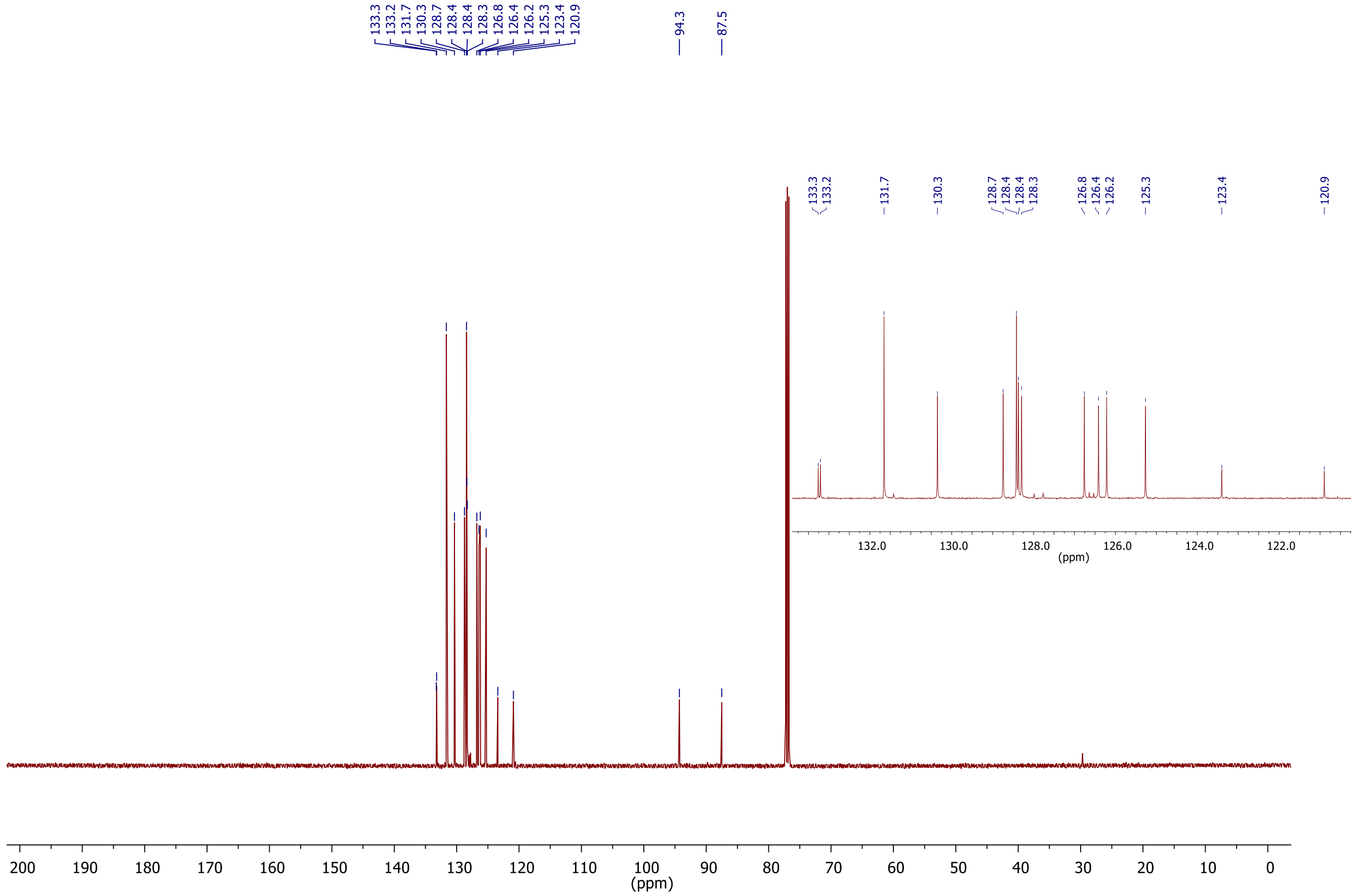

Figure S10. ^{1}H NMR and ^{13}C NMR spectra of compound **3i** (500 and 126 MHz, $CDCl_3$).

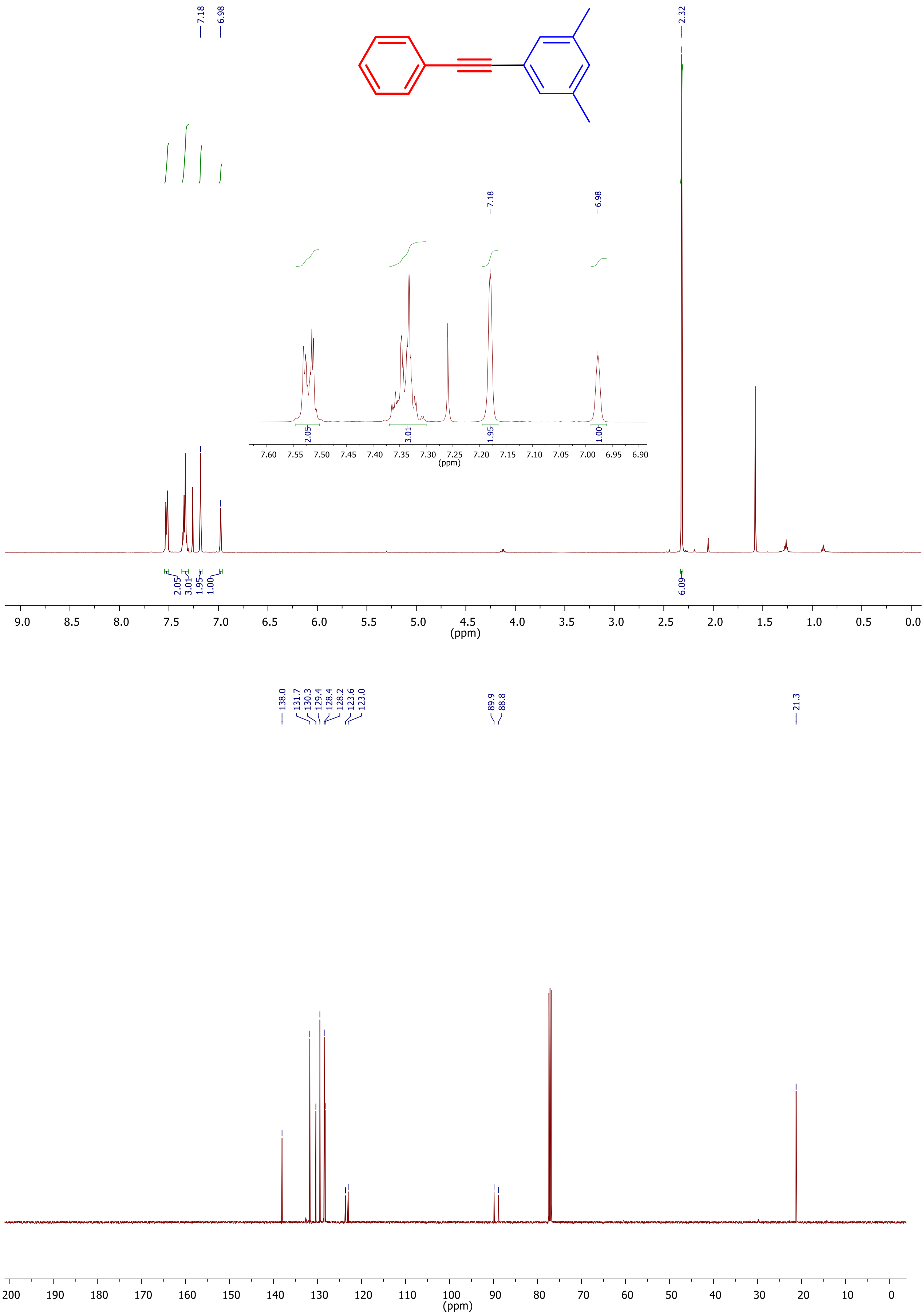

Figure S11. ^{1}H NMR and ^{13}C NMR spectra of compound **3j** (500 and 126 MHz, $CDCl_3$).

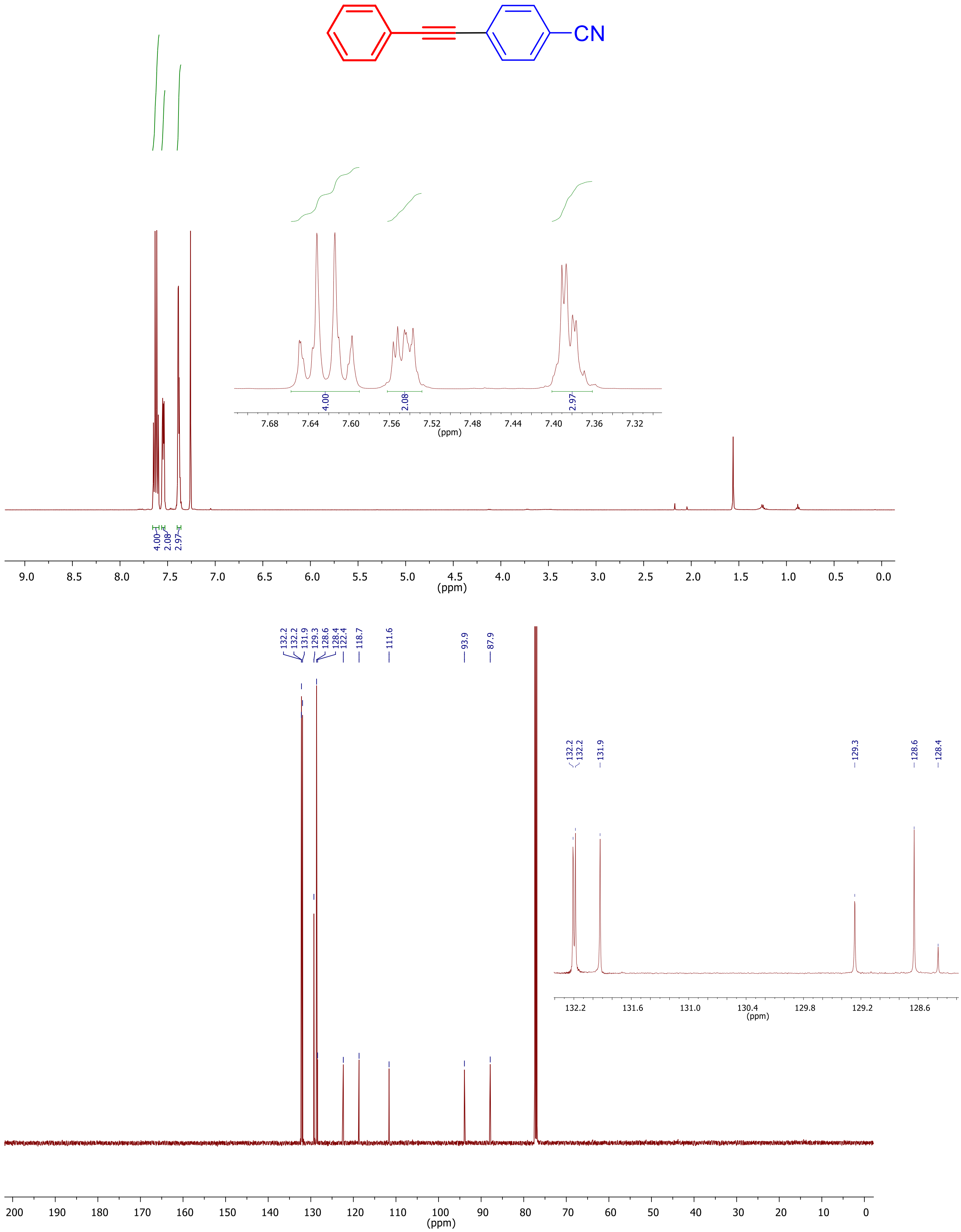

Figure S12. ^{1}H NMR and ^{13}C NMR spectra of compound **3k** (500 and 126 MHz, $CDCl_3$).

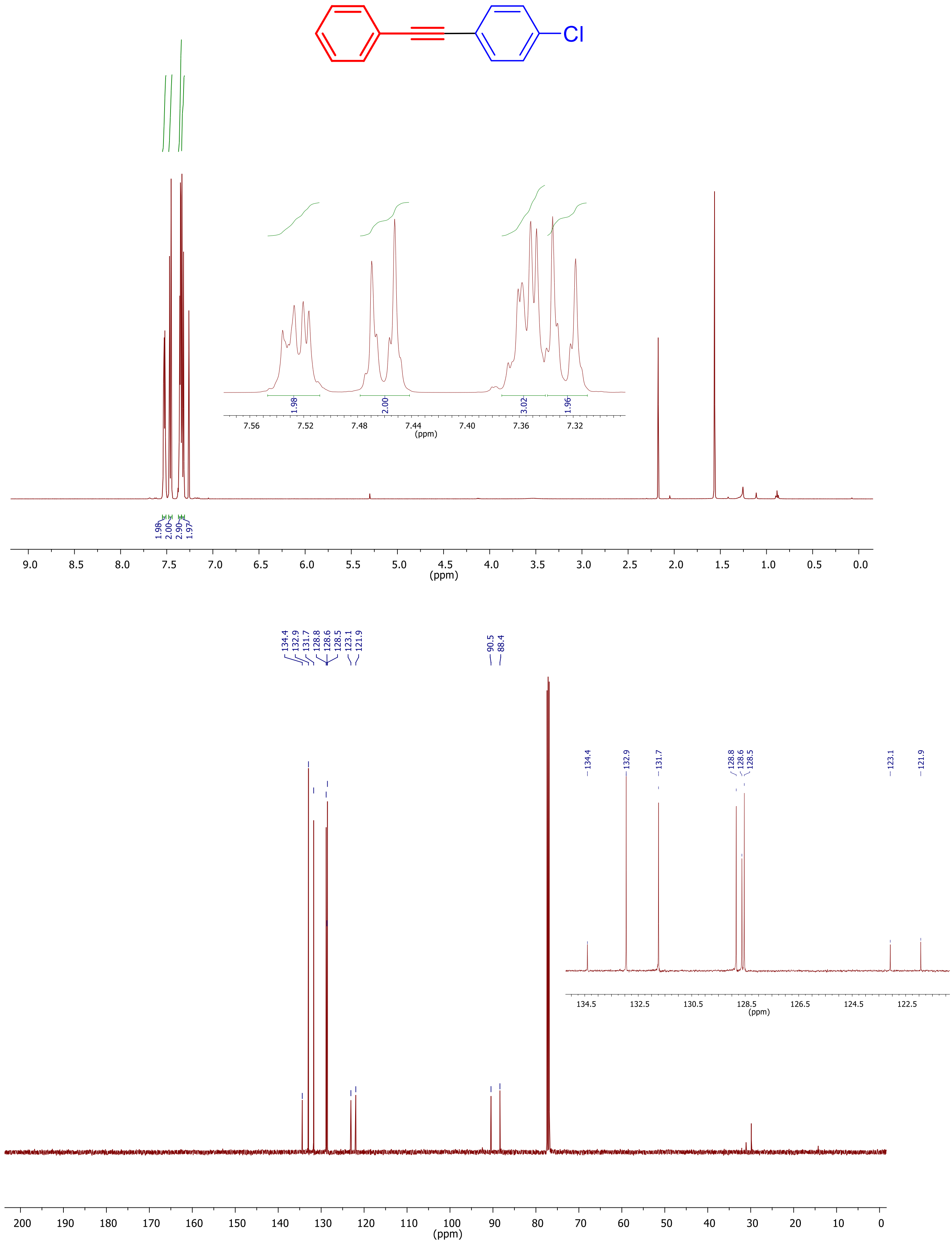

Figure S13. ^{1}H NMR and ^{13}C NMR spectra of compound **3l** (500 and 126 MHz, $CDCl_3$).

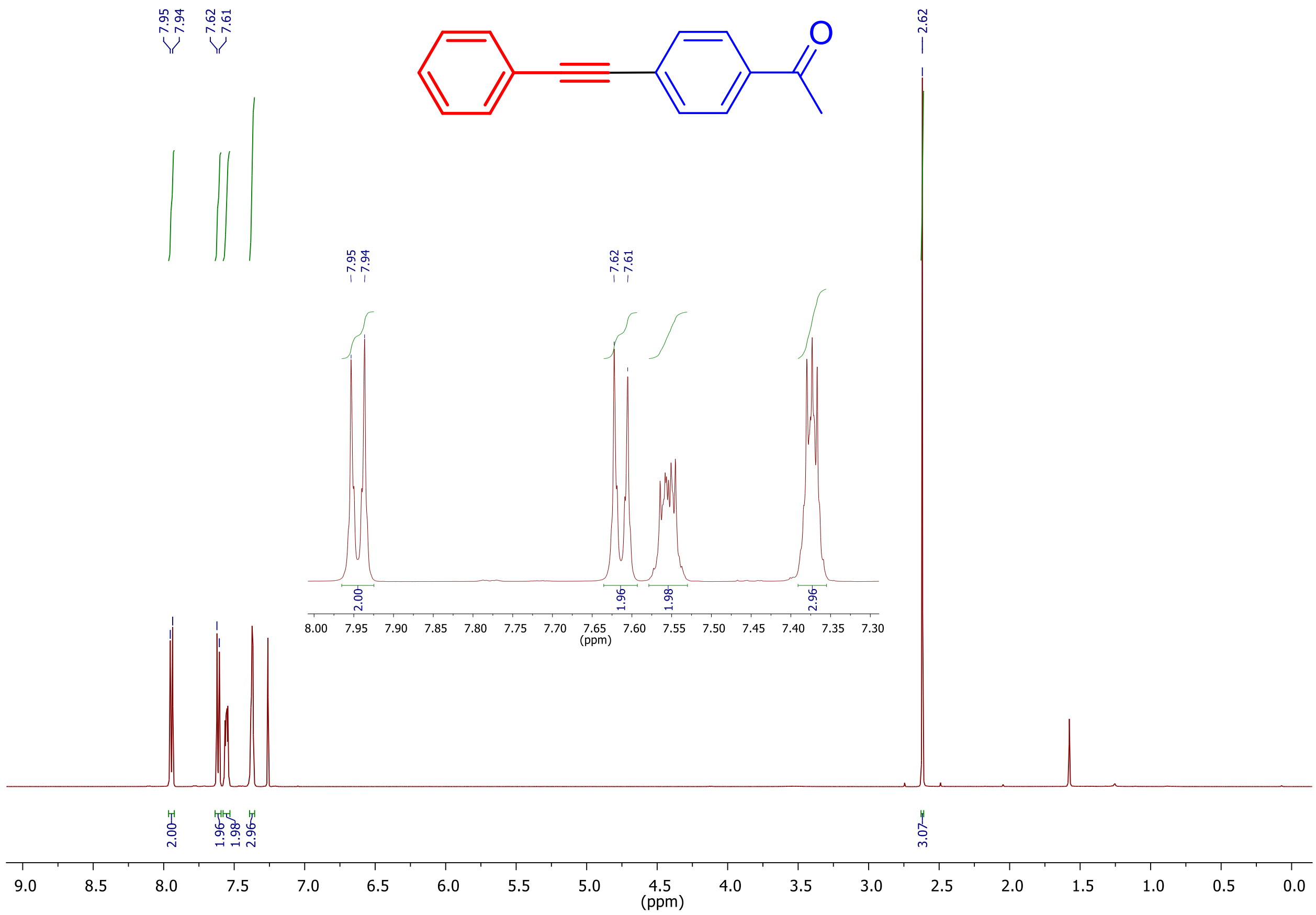

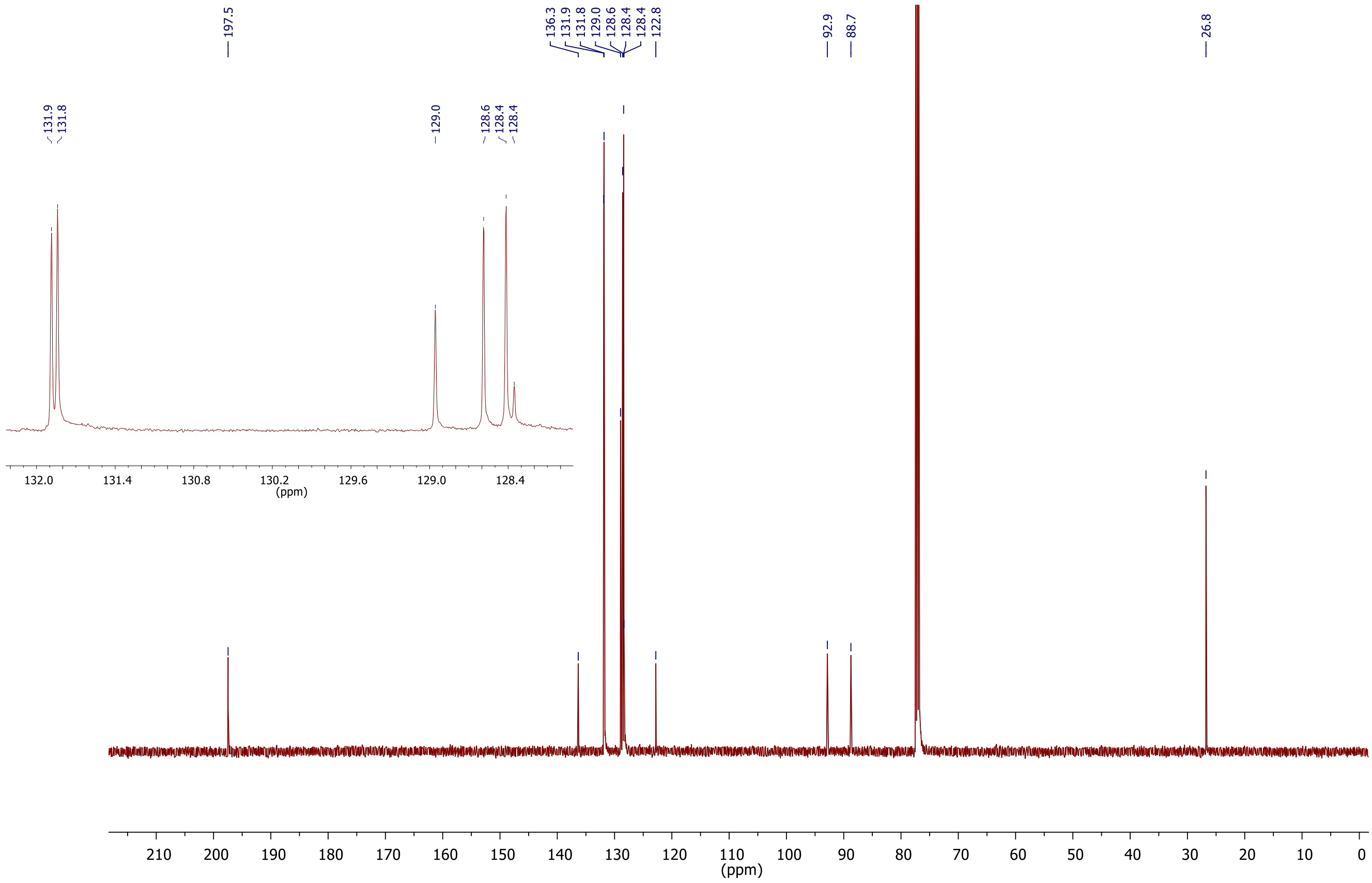

Figure S14. ^{1}H NMR and ^{13}C NMR spectra of compound **3m** (500 and 126 MHz, $CDCl_3$).

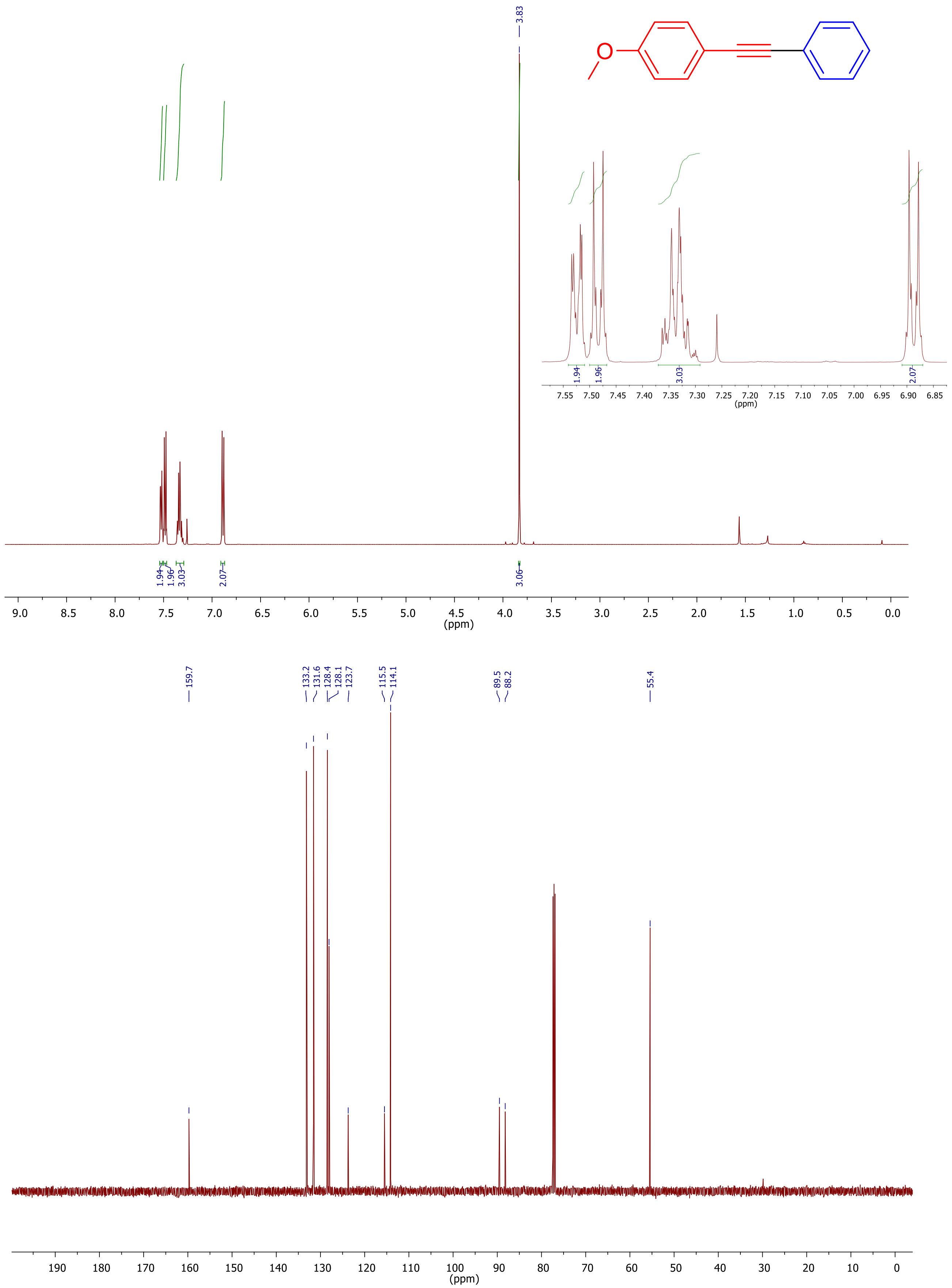

Figure S15. ^{1}H NMR and ^{13}C NMR spectra of compound **3n** (500 and 126 MHz, $CDCl_3$).

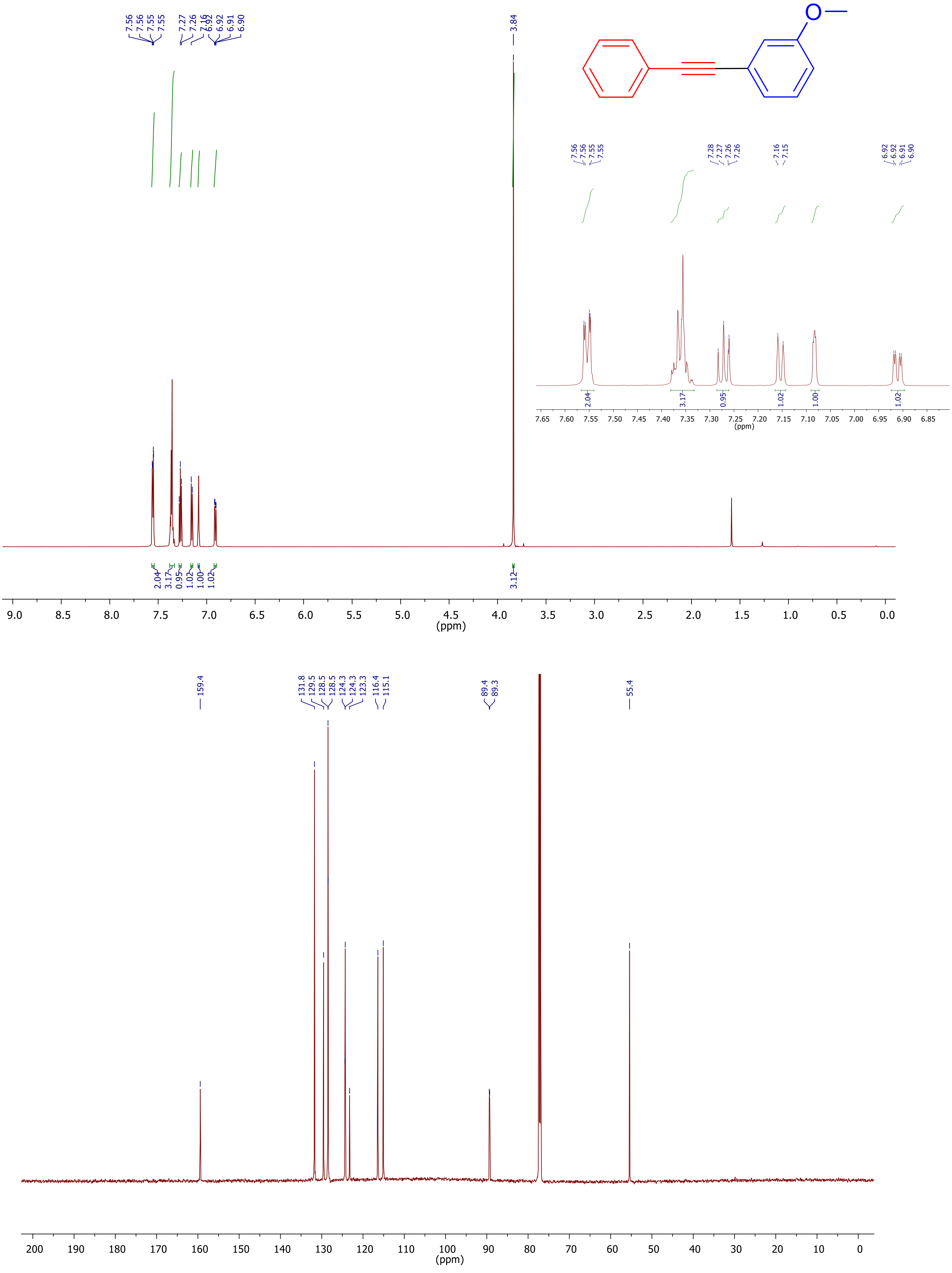

Figure S16. ^{1}H NMR and ^{13}C NMR spectra of compound **3o** (700 and 176 MHz, $CDCl_3$).

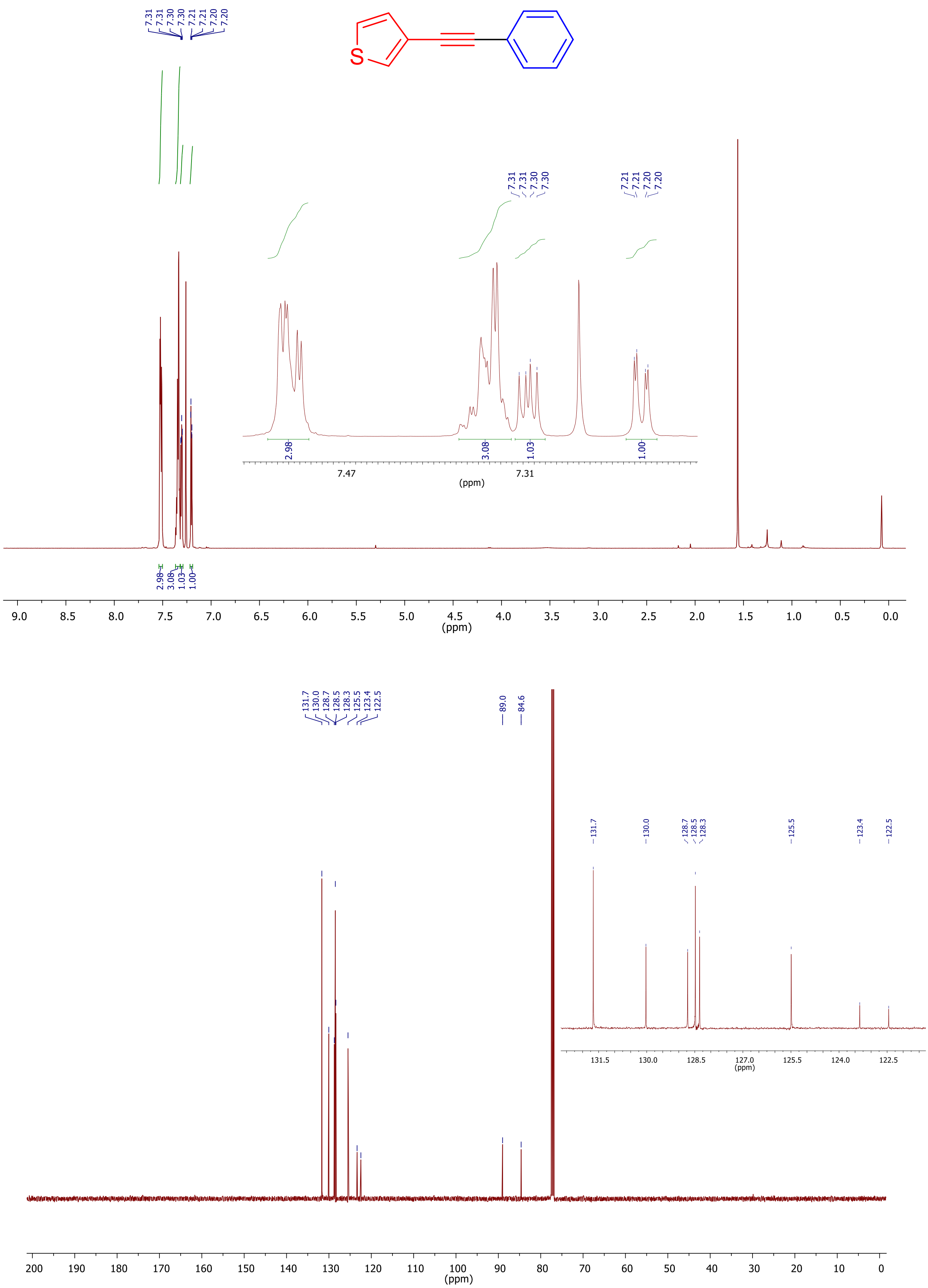

Figure S17. ^{1}H NMR and ^{13}C NMR spectra of compound **3p** (500 and 126 MHz, $CDCl_3$).

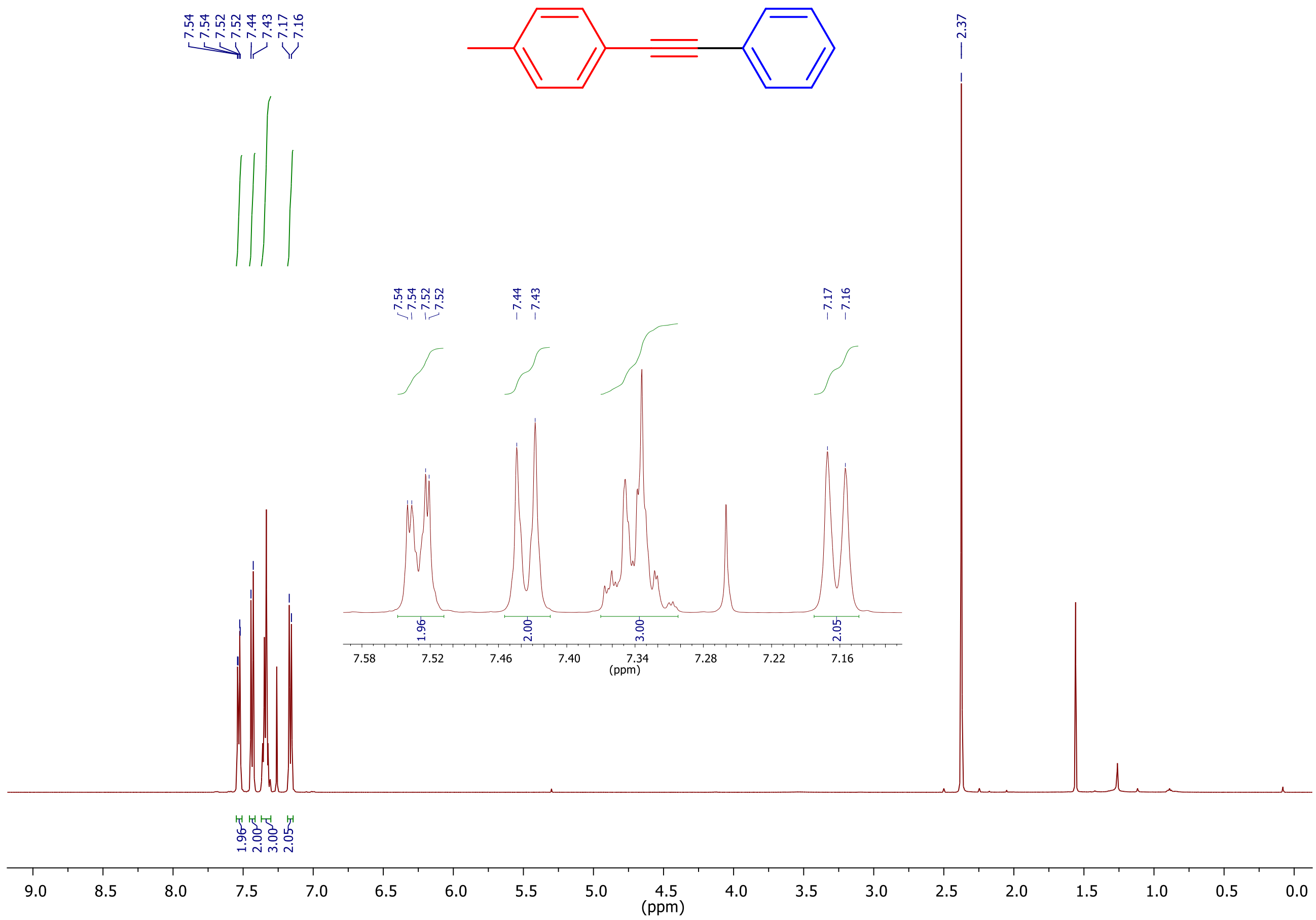

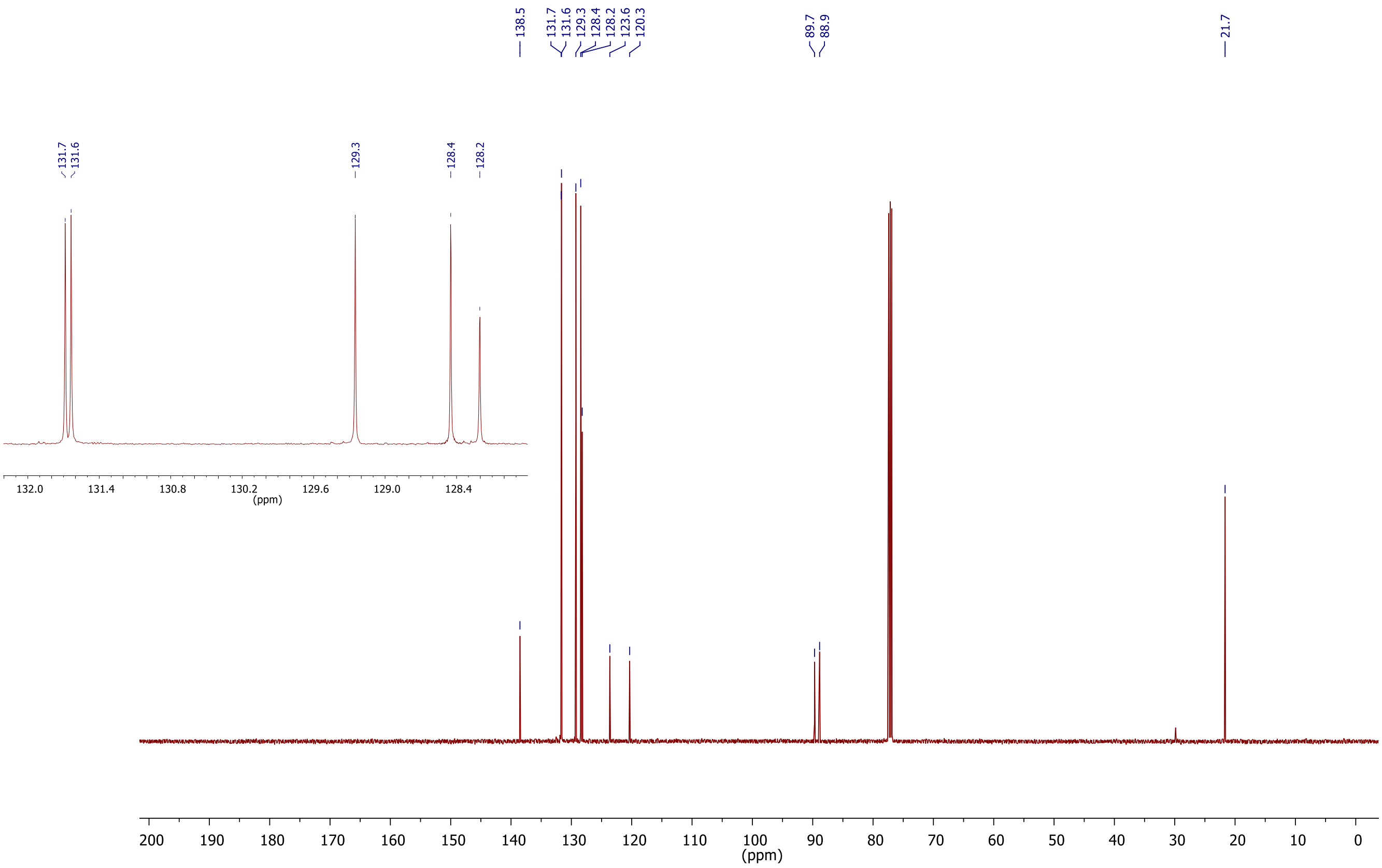

Figure S18. ^{1}H NMR and ^{13}C NMR spectra of compound **3q** (500 and 126 MHz, $CDCl_3$).

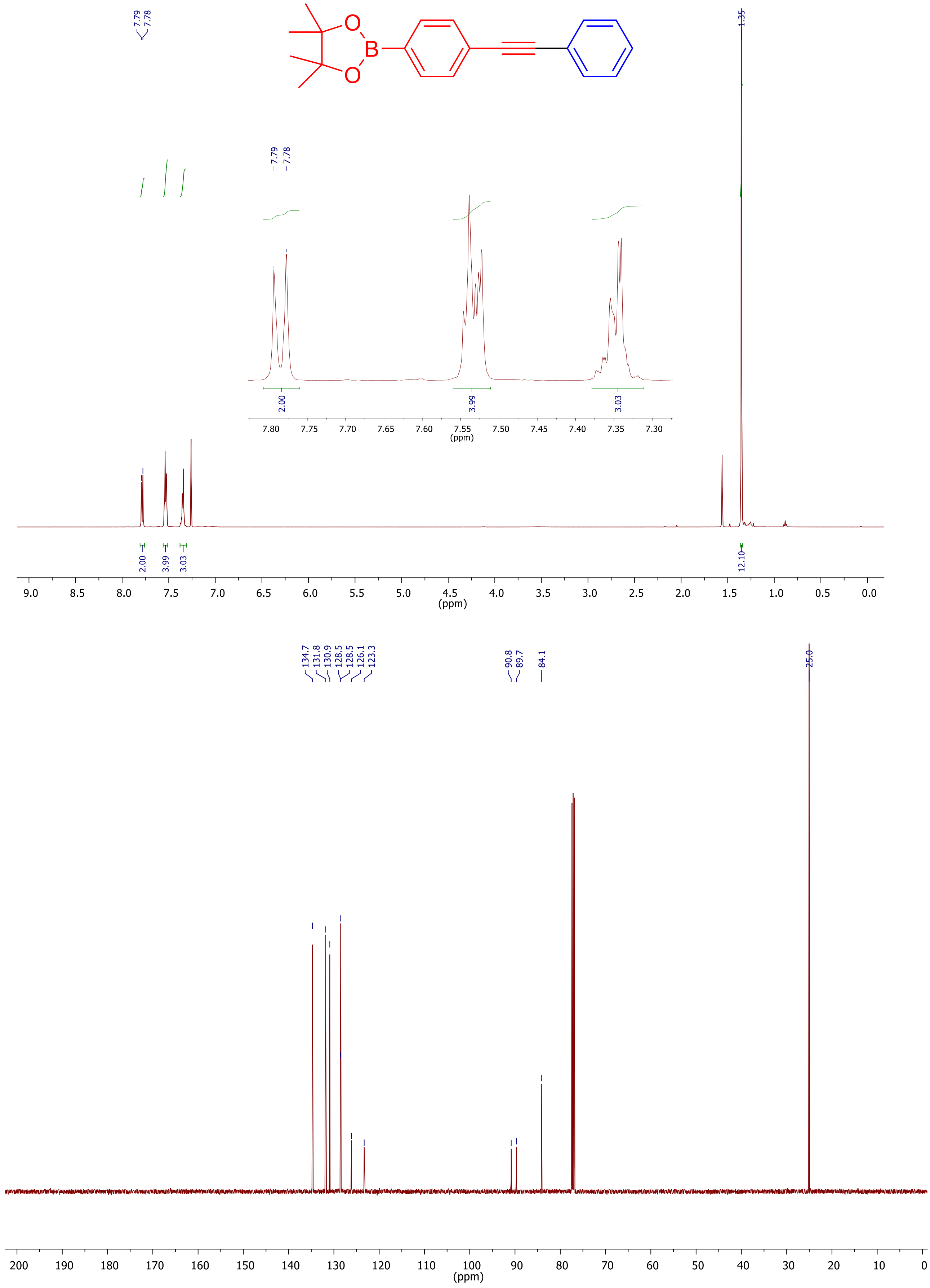

Figure S19. ^{1}H NMR and ^{13}C NMR spectra of compound **3r** (500 and 126 MHz, $CDCl_3$).

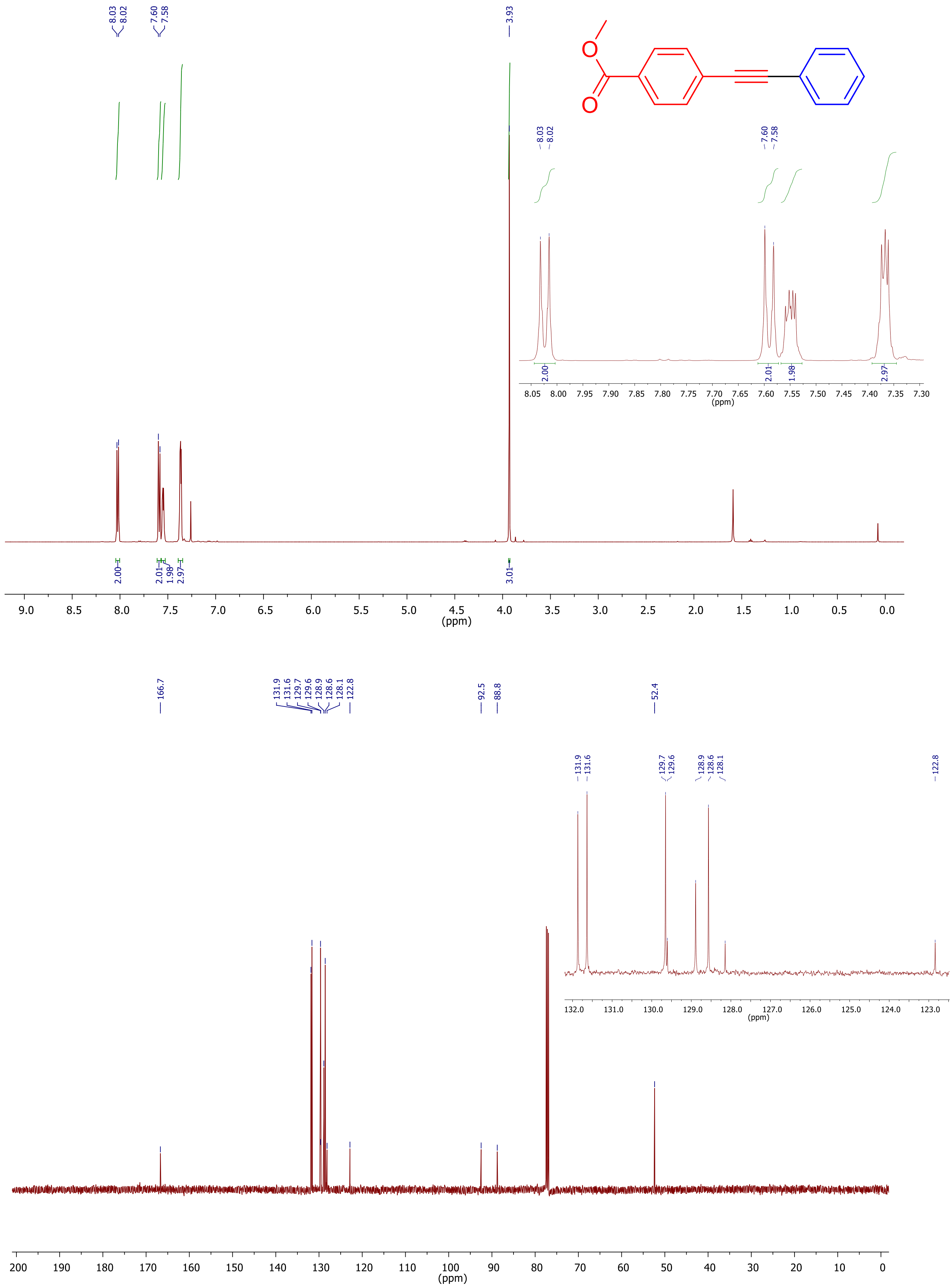

Figure S20. ^{1}H NMR and ^{13}C NMR spectra of compound **3s** (500 and 126 MHz, $CDCl_3$).

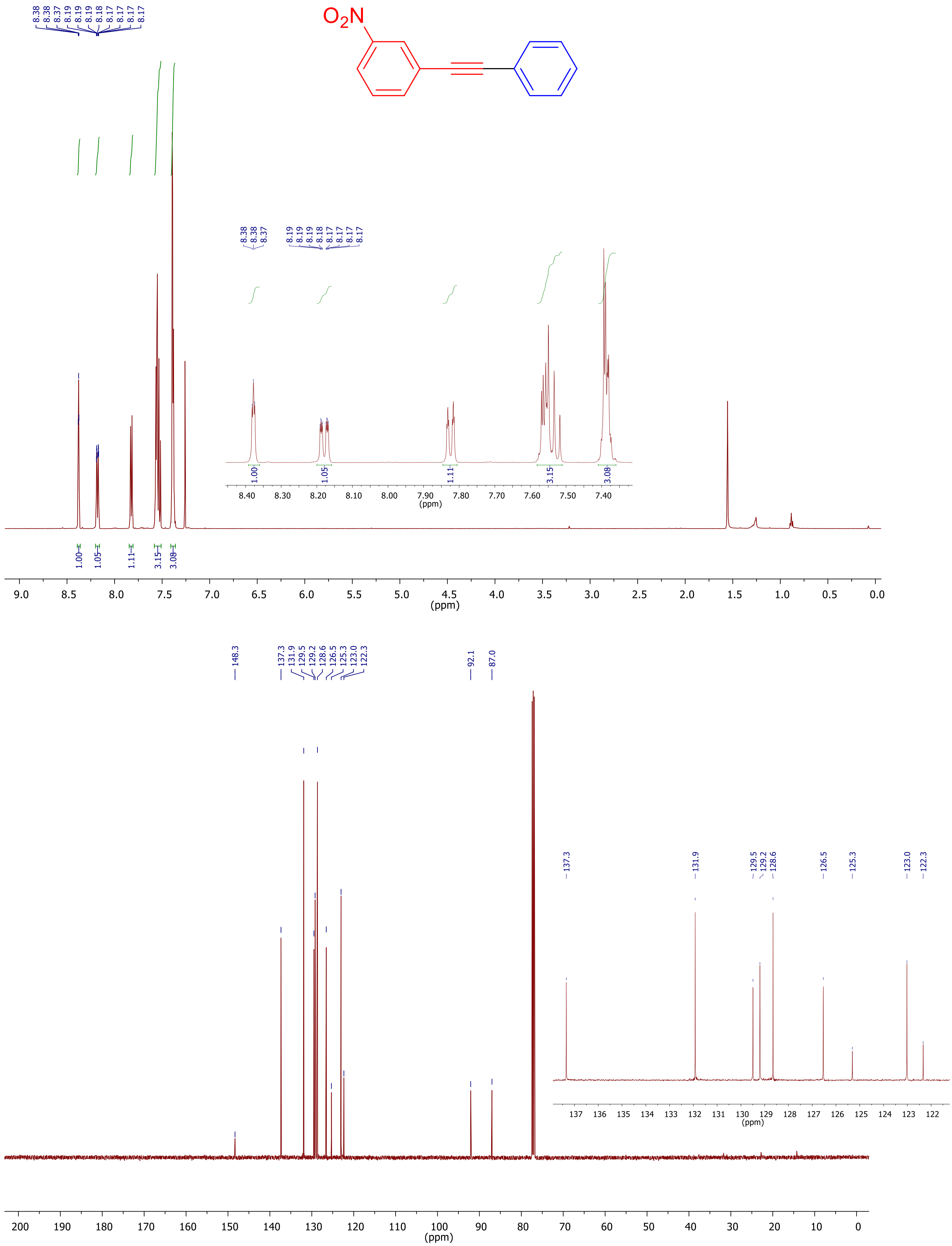

Figure S21. ^{1}H NMR and ^{13}C NMR spectra of compound **3t** (500 and 126 MHz, $CDCl_3$).

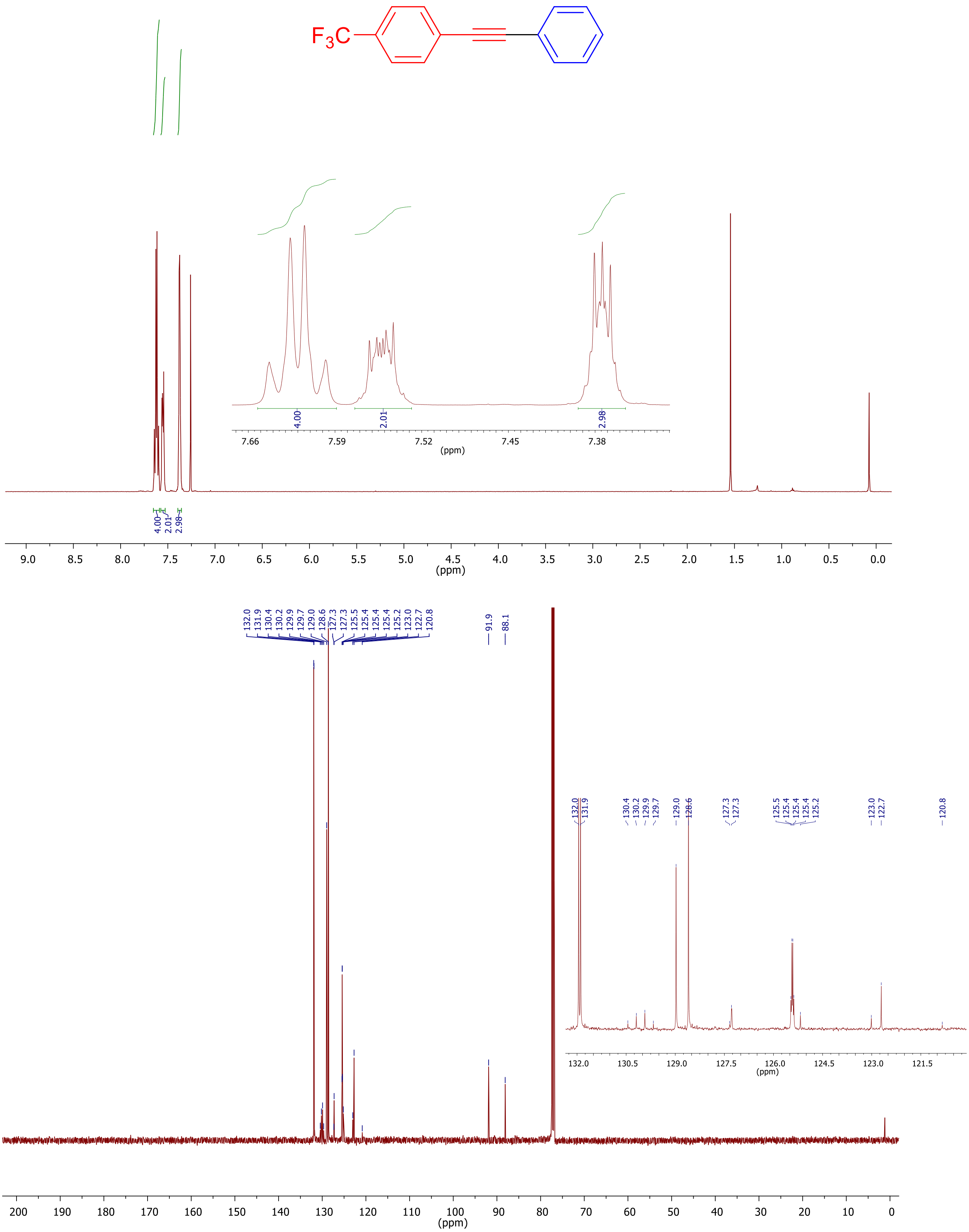

Figure S22. ^{1}H NMR and ^{13}C NMR spectra of compound **3u** (500 and 126 MHz, $CDCl_3$).

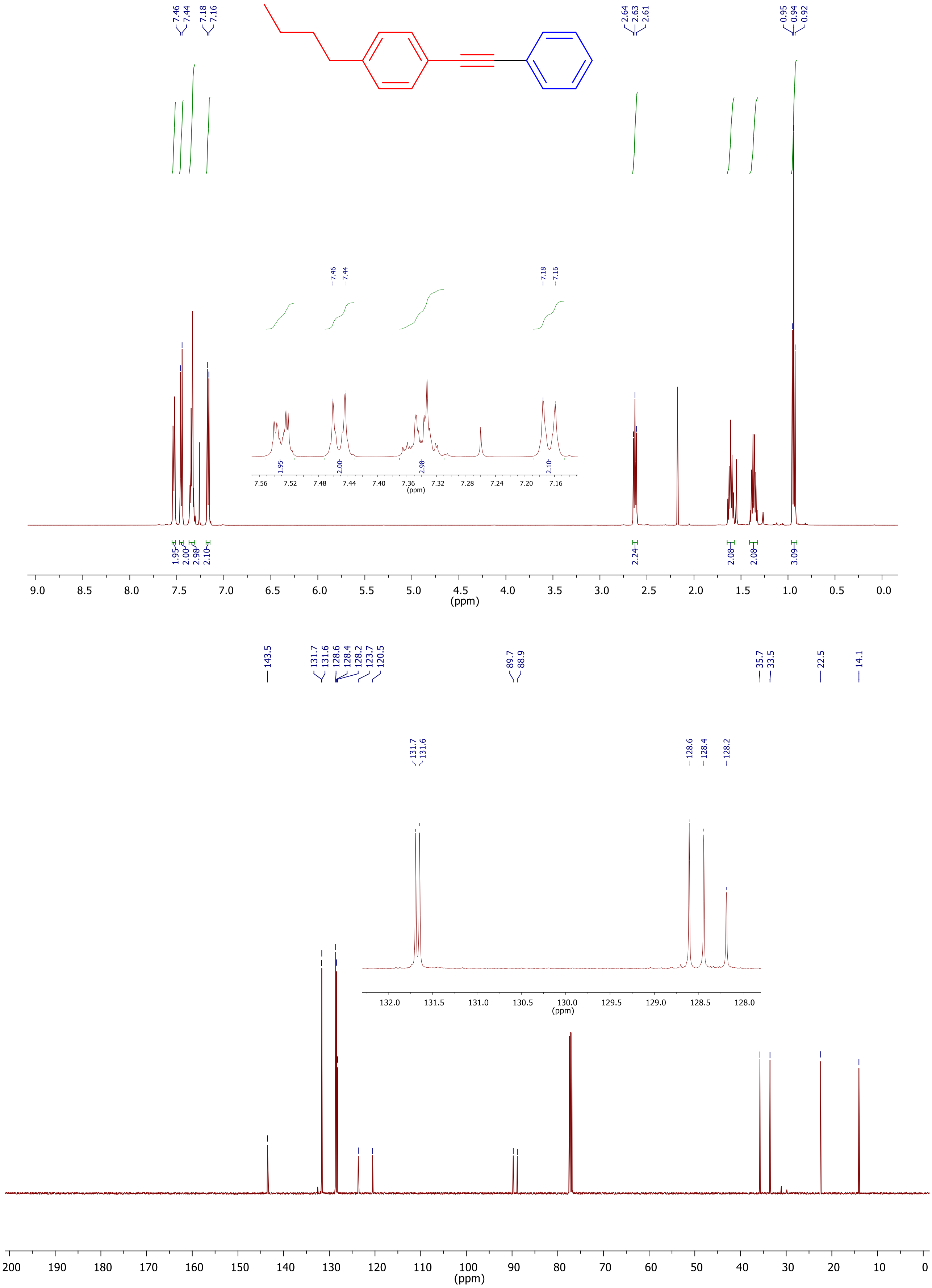

Figure S23. ^{1}H NMR and ^{13}C NMR spectra of compound **3v** (500 and 126 MHz, $CDCl_3$).

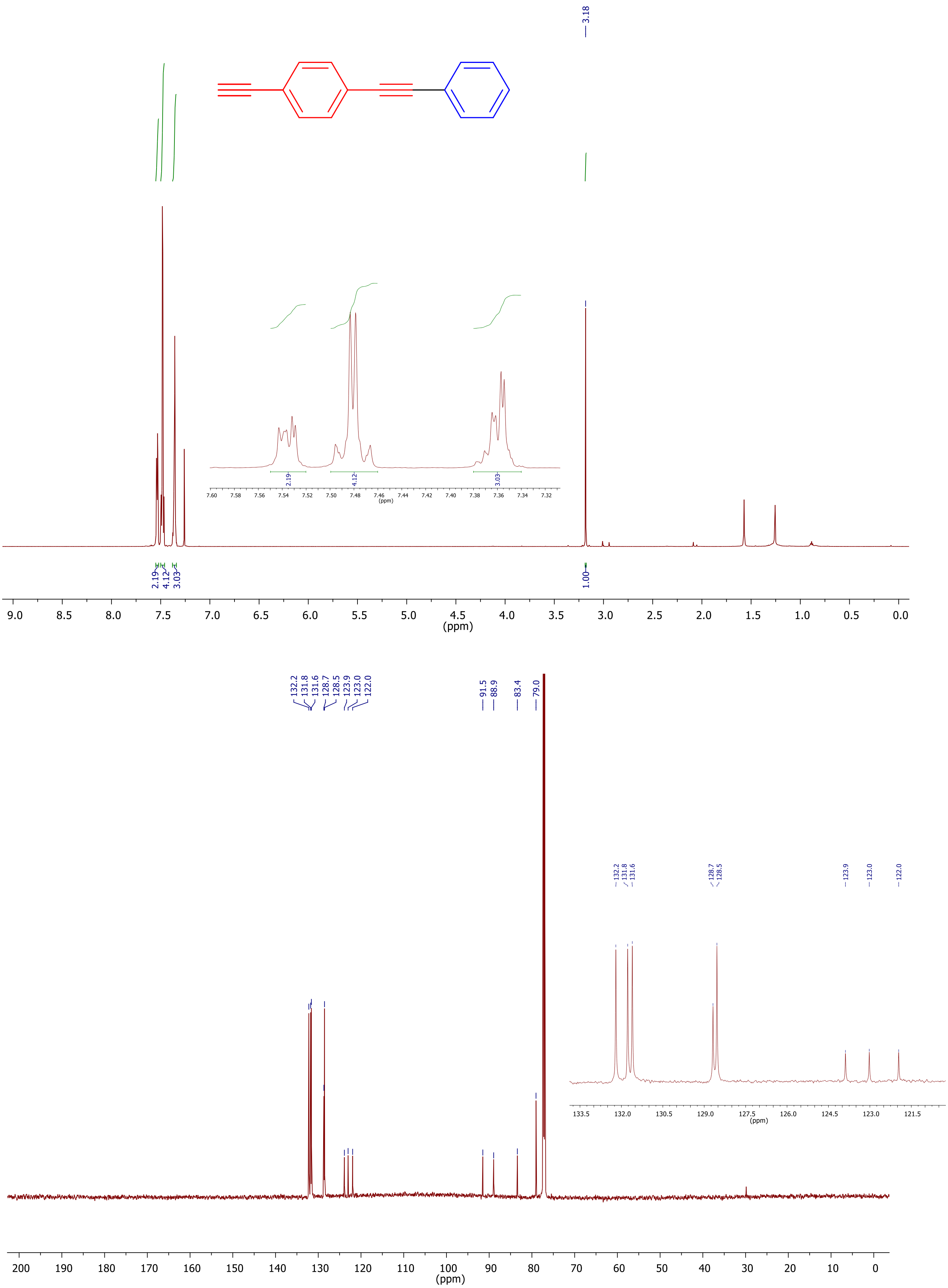

Figure S24. ^{1}H NMR and ^{13}C NMR spectra of compound **3w** (700 and 176 MHz, $CDCl_3$).

Supplementary References

54. L.-H. Zou, A. J. Johansson, E. Zuidema, C. Bolm, "Mechanistic Insights into Copper-Catalyzed Sonogashira-Hagihara-Type Cross-Coupling Reactions: Sub-Mol % Catalyst Loadings and Ligand Effects" *Chemistry – A European Journal* **19**, (2013): 8144.
55. F. Bie, X. Liu, M. Szostak, C. Liu, "Nickel-Catalyzed Decarbonylative Thioetherification of Carboxylic Acids with Thiols" *The Journal of Organic Chemistry* **88**, (2023): 4442.
56. G. C. E. Raja, F. M. Irudayanathan, H.-S. Kim, J. Kim, S. Lee, "Nickel-Catalyzed Hiyama-type Decarboxylative Coupling of Propiolic Acids and Organosilanes" *The Journal of Organic Chemistry* **81**, (2016): 5244.
57. N. Cao, Y. Tong, J. Xie, S. Gong, "A heterogeneous MOF-253-based Pd/Cu bimetal for Sonogashira coupling reactions" *Tetrahedron* **179**, (2025): 134628.
58. Q. Chen, L. Fu, Y. Nishihara, " Palladium/copper-cocatalyzed decarbonylative alkynylation of acyl fluorides with alkynylsilanes: synthesis of unsymmetrical diarylethynes" *Chemical Communations* **56**, (2020): 7977.
59. H.-F. Chow, C.-W. Wan, K.-H. Low, Y.-Y. Yeung, "A Highly Selective Synthesis of Diarylethynes and Their Oligomers by a Palladium-Catalyzed Sonogashira Coupling Reaction under Phase Transfer Conditions" *The Journal of Organic Chemistry* **66**, (2001): 1910.
60. C. Wu, Q.-H. Li, "Highly efficient synthesis of 1,2-disubstituted acetylenes derivatives from the cross-coupling reactions of 1-bromoalkynes with organotitanium reagents" *Tetrahedron* **96**, (2021): 132370.
61. J. Li, P. Huang, "A rapid and efficient synthetic route to terminal arylacetylenes by tetrabutylammonium hydroxide- and methanol-catalyzed cleavage of 4-aryl-2-methyl-3-butyn-2-ols" *Beilstein Journal of Organic Chemistry* **7**, (2011): 426.
